# BioHackathon 2015: Semantics of data for life sciences and reproducible research

**DOI:** 10.12688/f1000research.18236.1

**Published:** 2020-02-24

**Authors:** Rutger A. Vos, Toshiaki Katayama, Hiroyuki Mishima, Shin Kawano, Shuichi Kawashima, Jin-Dong Kim, Yuki Moriya, Toshiaki Tokimatsu, Atsuko Yamaguchi, Yasunori Yamamoto, Hongyan Wu, Peter Amstutz, Erick Antezana, Nobuyuki P. Aoki, Kazuharu Arakawa, Jerven T. Bolleman, Evan Bolton, Raoul J. P. Bonnal, Hidemasa Bono, Kees Burger, Hirokazu Chiba, Kevin B. Cohen, Eric W. Deutsch, Jesualdo T. Fernández-Breis, Gang Fu, Takatomo Fujisawa, Atsushi Fukushima, Alexander García, Naohisa Goto, Tudor Groza, Colin Hercus, Robert Hoehndorf, Kotone Itaya, Nick Juty, Takeshi Kawashima, Jee-Hyub Kim, Akira R. Kinjo, Masaaki Kotera, Kouji Kozaki, Sadahiro Kumagai, Tatsuya Kushida, Thomas Lütteke, Masaaki Matsubara, Joe Miyamoto, Attayeb Mohsen, Hiroshi Mori, Yuki Naito, Takeru Nakazato, Jeremy Nguyen-Xuan, Kozo Nishida, Naoki Nishida, Hiroyo Nishide, Soichi Ogishima, Tazro Ohta, Shujiro Okuda, Benedict Paten, Jean-Luc Perret, Philip Prathipati, Pjotr Prins, Núria Queralt-Rosinach, Daisuke Shinmachi, Shinya Suzuki, Tsuyosi Tabata, Terue Takatsuki, Kieron Taylor, Mark Thompson, Ikuo Uchiyama, Bruno Vieira, Chih-Hsuan Wei, Mark Wilkinson, Issaku Yamada, Ryota Yamanaka, Kazutoshi Yoshitake, Akiyasu C. Yoshizawa, Michel Dumontier, Kenjiro Kosaki, Toshihisa Takagi

**Affiliations:** 1 Institute of Biology Leiden, Leiden University, Leiden, The Netherlands; 2 Naturalis Biodiversity Center, Leiden, The Netherlands; 3 Database Center for Life Science, Tokyo, Japan; 4 Department of Human Genetics, Nagasaki University Graduate School of Biomedical Sciences, Nagasaki, Japan; 5 DDBJ Center, National Institute of Genetics, Mishima, Japan; 6 Shenzhen Institute of Advanced Technology, Chinese Academy of Sciences, Shenzhen, China; 7 Curoverse, Somerville, USA; 8 Department of Biology, Norwegian University of Science and Technology, Trondheim, Norway; 9 Faculty of Science and Engineering, SOKA University, Tokyo, Japan; 10 Institute for Advanced Biosciences, Keio University, Tokyo, Japan; 11 SIB Swiss Institute of Bioinformatics, Centre Medical Universitaire, Lausanne, Switzerland; 12 National Center for Biotechnology Information, National Library of Medicine, National Institutes of Health, Bethesda, USA; 13 Istituto Nazionale Genetica Molecolare, Romeo ed Enrica Invernizzi, Milan, Italy; 14 Dutch Techcentre for Life Sciences, Utrecht, The Netherlands; 15 National Institute for Basic Biology, National Institutes of Natural Sciences, Okazaki, Japan; 16 Computational Bioscience Program, University of Colorado School of Medicine, Denver, USA; 17 Université Paris-Saclay, LIMSI, CNRS, Paris, France; 18 Institute for Systems Biology, Seattle, USA; 19 Universidad de Murcia, IMIB-Arrixaca, Murcia, Spain; 20 National Institute of Genetics, Mishima, Japan; 21 RIKEN Center for Sustainable Resource Science, Yokohama, Japan; 22 Polytechnic University of Madrid, Madrid, Spain; 23 Research Institute for Microbial Diseases, Osaka University, Osaka, Japan; 24 St Vincent's Clinical School, Faculty of Medicine, University of New South Wales, Darlinghurst, Australia; 25 Kinghorn Centre for Clinical Genomics, Garvan Institute of Medical Research, Darlinghurst, Australia; 26 Novocraft Technologies Sdn. Bhd., Selangor, Malaysia; 27 Computational Bioscience Research Center, King Abdullah University of Science and Technology, Thuwal, Saudi Arabia; 28 European Molecular Biology Laboratory, European Bioinformatics Institute, Hinxton, UK; 29 Institute for Protein Research, Osaka University, Osaka, Japan; 30 School of Life Science and Technology, Tokyo Institute of Technology, Tokyo, Japan; 31 The Institute of Scientific and Industrial Research, Osaka University, Osaka, Japan; 32 Hitachi Ltd., Tokyo, Japan; 33 National Bioscience Database Center, Japan Science and Technology Agency, Tokyo, Japan; 34 Institute of Veterinary Physiology and Biochemistry, Justus-Liebig University Giessen, Giessen, Germany; 35 Gesellschaft für innovative Personalwirtschaftssysteme mbH (GIP GmbH), Offenbach, Germany; 36 The Noguchi Institute, Tokyo, Japan; 37 National Cancer Center Japan, Tokyo, Japan; 38 National Institutes of Biomedical Innovation, Health and Nutrition, Osaka, Japan; 39 Center for Information Biology, National Institute of Genetics, Mishima, Japan; 40 Lawrence Berkeley National Laboratory, Berkeley, USA; 41 RIKEN Quantitative Biology Center, Osaka, Japan; 42 Department of Systems Science, Osaka University, Osaka, Japan; 43 Tohoku Medical Megabank Organization, Tohoku University, Sendai, Japan; 44 Niigata University Graduate School of Medical and Dental Sciences, Niigata, Japan; 45 UC Santa Cruz Genomics Institute, University of California, Santa Cruz, USA; 46 INVENesis, Neuchâtel, Switzerland; 47 University Medical Center Utrecht, Utrecht, The Netherlands; 48 University of Tennessee Health Science Center, Memphis, USA; 49 Department of Biomedical Informatics, Harvard Medical School, Boston, Massachusetts, USA; 50 Graduate School of Pharmaceutical Sciences, Kyoto University, Kyoto, Japan; 51 RIKEN BioResource Center, Ibaraki, Japan; 52 Leiden University Medical Center, Leiden, The Netherlands; 53 WurmLab, School of Biological & Chemical Sciences, Queen Mary University of London, London, UK; 54 Escuela Técnica Superior de Ingeniería Agronómica, Alimentaria y de Biosistemas, Universidad Politécnica de Madrid, Madrid, Spain; 55 Oracle Corporation, Tokyo, Japan; 56 Graduate School of Agricultural and Life Sciences, The University of Tokyo, Tokyo, Japan; 57 Institute of Data Science, Maastricht University, Maastricht, The Netherlands; 58 Center for Medical Genetics, Keio University School of Medicine, Tokyo, Japan; 59 Department of Biological Sciences, Graduate School of Science, The University of Tokyo, Tokyo, Japan

**Keywords:** BioHackathon, Bioinformatics, Semantic Web, Web Services, Ontology, Visualization, Databases, Linked Open Data, Metadata, Workflows

## Abstract

We report on the activities of the 2015 edition of the BioHackathon, an annual event that brings together researchers and developers from around the world to develop tools and technologies that promote the reusability of biological data. We discuss issues surrounding the representation, publication, integration, mining and reuse of biological data and metadata across a wide range of biomedical data types of relevance for the life sciences, including chemistry, genotypes and phenotypes, orthology and phylogeny, proteomics, genomics, glycomics, and metabolomics. We describe our progress to address ongoing challenges to the reusability and reproducibility of research results, and identify outstanding issues that continue to impede the progress of bioinformatics research. We share our perspective on the state of the art, continued challenges, and goals for future research and development for the life sciences Semantic Web.

## Abbreviations

### Miscellaneous

API, Application Programming Interface; BH15, BioHackathon 2015; CUI, Concept Unique Identifier; CV, Controlled Vocabulary; DOID, DO IDentifier; DPA, Disease-Phenotype Association; EHR, Electronic Health Records; FAIR, Findable, Accessible, Interoperable and Reusable; GDA, Gene-Disease Association; GPM, General Process Model; LIMS, Laboratory Information Management System; MSEA, Metabolite Set Enrichment Analysis; ORCID, Open Researcher and Contributor ID; NLP, Natural Language Processing; NMR, Nuclear Magnetic Resonance; VG, genomic Variation Graph.

### Ontologies and vocabularies

BAO, BioAssay Ontology; CDAO, Comparative Data Analysis Ontology; ChEBI, Chemical Entities of Biological Interest; CHEMINF, CHEMical INFormation ontology; DC, DCT, Dublin Core, Dublin Core Terms; DO, Disease Ontology; EFO, Experimental Factor Ontology; EpSO, Epilepsy and Seizure Ontology; ERO, Eagle-i Resource Ontology; EXACT, Experiment ACTions ontology; EXPO, Ontology of scientific experiments; FMA, Foundational Model of Anatomy; FOAF, Friend Of A Friend; GO, Gene Ontology; HPO, Human Phenotype Ontology; IAO, Information Artifact Ontology; LABORS, LABoratory Ontology for Robot Scientists; MOD, Metadata for Ontology Description; MP, Mammalian Phenotype ontology; OA, Open Annotation ontology; OBAN, Ontology of Biomedical AssociatioN; OBI, Ontology for Biomedical Investigations; OMV, Ontology Metadata Vocabulary; ORDO, Orphanet Rare Disease Ontology; ORTH, ORTHology ontology; PATO, Phenotypic quality ontology; PICO, Patient Intervention Comparison Outcome; PIERO, Partial Information of chemical transformation; RO, Relations Ontology; SIO, Semanticscience Integrated Ontology; SIRO, Sample, Instrument, Reagent, Objective; SMART Protocols, SeMAntic RepresenTation for experimental protocols; UMLS, Unified Medical Language System.

### Organizations

BTMG, Biomedical Text Mining Group at the NIH; DBCLS, Database Center for Life Science; EBI, European Bioinformatics Institute; GA4GH, Global Alliance for Genomics and Health; HGNC, HUGO Gene Nomenclature Committee; jPOST, Japan Proteome Standard Repository/Database; LOV, Linked Open Vocabularies; NBDC, National Bioscience Database Center; NCBI, National Center for Biotechnology Information; NCBO, National Center for Biomedical Ontology; NESCent, National Evolutionary Synthesis Center; NIH, National Institutes of Health; OBO Foundry, Open Biomedical Ontologies Foundry; Open PHACTS, Open Pharmacological Concept Triple Store; PDBj, Protein Database Japan; RDA, Research Data Alliance.

### Project

CWL, Common Workflow Language; DisGeNET, Disease Gene Network; GEO, Gene Expression Omnibus; HUPO-PSI, Human Proteome Organization Proteomics Standards Initiative; KEGG-OC, Kyoto Encyclopedia of Genes and Genomes – Orthologous Clusters; LSDB Archive, Life Science Database Archive; MBGD, Microbial Genome Database; MeKO, Metabolite profiling database for Knock-Out mutants in Arabidopsis; OLS, Ontology Lookup Service; OMA, Orthologous MAtrix; OMIM, Online Mendelian Inheritance in Man; ORKA, Open, Reusable Knowledge graph Annotator; PASSEL, Peptide AtlaS SRM Experiment Library; PMR, Plant and Microbial Metabolomics Resource; PRIDE, PRoteomics IDEntifications database ; QfO, Quest for Orthologs; SADI, Semantic Automated Discover and Integration; SIDER, SIDe Effect Resource; SWIT, Semantic Web Integration Tool.

### Technologies

BED, Browser Extensible Data; HPC, High Performance Computing; HTTP, HyperText Transfer Protocol; JSON, JavaScript Object Notation; JSON-LD, JSON – Linked Data; LOD, Linked Open Data; OWL, Web Ontology Language; RDF, Resource Description Framework; RDFa, RDF in Attributes; RML, RDF Modeling Language; SAM/BAM, Sequence Alignment/Map, Binary Alignment/Map; SHA, Secure Hash Algorithm; SPARQL, SPARQL Protocol and RDF Query Language; TPF, Triple Pattern Fragments ^1^; URI, Universal Resource Identifier; VCF, Variant Call Format; YAML, YAML Ain’t Markup Language; XML, eXtensible Markup Language.

## Background

The past few years have yielded considerable progress in the development and application of fundamental digital technologies that support research in the life sciences ^2^, including ontologies and Linked Open Data (LOD), semantic web services, natural language processing, and tooling for workflows and virtualization. While these technologies are useful for life sciences research, key to their long-term success lies in community agreements that foster standardization and interoperability ^2^. In an effort to coordinate the social and technological aspects of *in silico* life sciences research, the authors convened at the 2015 edition of the BioHackathon (BH15), an event that aims to create a highly collaborative environment to explore, evaluate, and implement solutions to the problems of data publication, integration, and reuse ^3– 6^. A hackathon is a type of software development lifecycle model featuring problem-focused development via intensive, time-limited, self-organized group activities, typically involving programmers and various types of collaborators ^7^. The hackathon methodology has been shown to be productive in a variety of biomedical fields, including rehabilitative healthcare ^8^, biological data science ^9^, neuroscience ^10^, computer-aided differential diagnosis ^11^, stroke detection ^12^, standards specification in systems and synthetic biology ^13^, data science for knowledge discovery in medicine ^14^, medical device innovation ^15^, enrichment of biodiversity data ^16^, and teaching genomics ^17^. BH15 was held in Nagasaki, Japan, over the period of September 14 ^th^ to 18 ^th^ 2015, and was hosted by the National Bioscience Database Center (NBDC, ^18^) and the Database Center for Life Science (DBCLS, ^19^) to promote interoperability of life sciences databases in Japan. Researchers and developers from around the world participated by invitation. BH15 was preceded by a public symposium featuring new research and updates from the participants. BH15 involved 80 individuals from 12 countries and a wide variety of backgrounds, including computer programmers, bioinformaticians, biocurators, ontologists, biological scientists, systems biologists, data scientists, and linguists.

**Figure 1.**
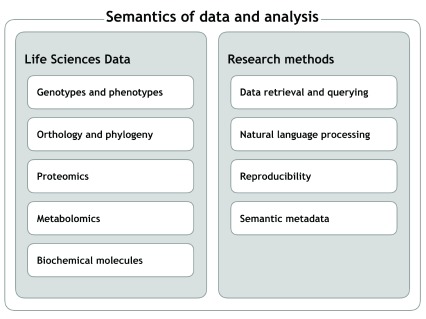
Main themes and topics of the BioHackathon 2015.

Here, we present selected outcomes from BH15, self-organized by the participants in projects around different topics, which we discuss in the following sections. At the highest level, the contours of these topics are, broadly, i) *life sciences data*, including genotypes and phenotypes, orthology and phylogeny, proteomics, metabolomics, and biochemical molecular; and ii) *research methods*, i.e. the technologies that support *in silico* analysis in the life sciences, including data retrieval and querying, natural language processing, reproducibility, and semantic metadata. Under these broad topics, we identify various themes within which specific activities took place. These topics and themes are illustrated in Figure 1. The activities and their scopes were identified by the participants through self-organization following Open Space technology ^20^. As such, the commitment of the participants to any particular activity was somewhat free-wheeling, and so we report the outcomes collectively, rather than subdivided by participant teams. The results of the work reported here are relevant both to evaluate the current state of the relevant technologies and problem areas in the life sciences, and to help the field understand the potential and problems of future research and development efforts.

## Life sciences data

### Genotypes and phenotypes

***Variation graph construction.*** In the context of the Global Alliance for Genomics and Health (GA4GH, ^21^) there is a challenge to build genomic variation graphs (VG). A genomic variation graph represents all “common” genetic variation, providing a means to stably name and canonically identify each variant. At BH15, we modeled such graphs using RDF semantics. Taking the 1000 Genomes project phase 3 Variant Call Format (VCF) files and the GRCh37 human reference genome we built a variant graph using the VG tool ^22^. Such a VG graph corresponds to just fewer than 2 billion triples. It was loaded inside 67 minutes on a server from 2013 that had 64 AMD X86_64 cores, 256 GB ram, and 3 TB of consumer-quality SSD storage without specific tuning. The SPARQL database disk footprint with indexes was 49 GB, i.e. double the disk space consumed by the raw VG tool files. This shows that a modern SPARQL database does not require exorbitant resources to be able to index and load a variant graph of interest to the medical community. We also demonstrated that a number of queries on the graph executed within reasonable times. This work was contributed to the VG development team and incorporated into the VG release 1.4.0. At BH15, the standard API developed by the core API team of the GA4GH was implemented as a service running on top of a SPARQL endpoint.

***Variant call transformation.*** VCF is a standard for text files that store gene sequence variations and is used for large-scale genotyping and DNA sequencing projects. Converting a single high-throughput sequence dataset, e.g. a VCF file but more so a very large database such as the Ensembl Variation Database, into RDF results in a number of triples that may be unmanageable for a small bioinformatics lab, even if backed by current hardware. However, data can also be considered in a more dynamic way, if we abstract the concept of data generation to, for instance, some bioinformatics analysis or pipeline, where new data can be generated on the fly as a result of some computation over existing information or files. To this end we prototyped a real-time system to transform VCF into RDF and query it by SPARQL. With JRuby we could use the original samtools/htsjdk libraries for manipulating VCF, BED, SAM/BAM files. With this approach, we could quickly prototype our solution and defer the development of proper Java libraries sharable by alternative approaches and/or applications. Our approach allows generating virtual endpoints over multiple VCF files, combining the simplicity of native file formats with the power of the SPARQL language, significantly improving the way we link and query heterogeneous information. An implementation of such a system was conceived during the 1 ^st^ RDF summit in 2014 at the DBCLS in Tokyo and further developed during BH15. The system ^23^ was based on *de facto* standard frameworks, such as Ruby RDF ^24^ and OpenSesame ^25^, which facilitate the generation and transformation of RDF based data and the processing of SPARQL algebra and queries.

***Phenotype ontology translation.*** Precision medicine aims to provide patient-tailored diagnostics, prognostics, treatments, and prevention. Part of the strategy to precision medicine involves more precise clinical phenotyping. The Human Phenotype Ontology (HPO) is an ontology of abnormal phenotypes associated with human disease ^26^. Originally aimed to describe Mendelian genetic diseases, it has since been expanded to cover phenotypes associated with rare and common diseases. The availability of phenotype terms expressed in the Japanese language is key to its application in text mining Electronic Health Records (EHR) in Japan.

The development project of HPO-Japanese was initiated prior to BH15 in cooperation with the HPO teams (Dr. Peter Robinson, Dr. Melissa Haendel, Dr. Nicole Vasilevsky, and Dr. Tudor Groza). We translated English terms into Japanese by exact matches to existing English-Japanese dictionaries, including the Elements of Morphology – Standard Terminology (Japanese ed.), the Japanese Association of Medical Sciences Medical Term Dictionary, the Life Science Dictionary (LSD), and general dictionaries. The total number of terms translated is 11,425. Elements of Morphology – Standard Terminology (Japanese ed.) covers ~400 terms (3.5%), the Japanese Association of Medical Sciences Medical Term Dictionary covers 1,807 terms (15.8%). The remaining terms need to be curated by experts. We are now compiling several translated terms as curated HPO-Japanese. Once completed, HPO-Japanese will be open and available so that precise and standardized phenotyping can be undertaken using Japanese EHR text and which can be directly linked to the international resources and research systems through HPO identifiers.

### Orthology and phylogeny

***Orthology ontology development and application.*** Orthologs are defined as genes derived from a common ancestral gene by speciation. Orthology information can play a central role in predicting gene function in newly sequenced genomes and can also help unravel the evolutionary history of genes and organisms. Orthology resources have been represented in a variety of formats, including the OrthoXML ^27^ that is used by several orthology databases such as InParanoid ^28^, Orthologous MAtrix (OMA, ^29^), and TreeFam ^30^. The interest in exchanging orthology data with other communities has provided the impetus for research on applying the Semantic Web and using common ontologies for making the meaning of the content explicit. Thus, on the basis of previous studies on the semantic representation of orthology ^31, 32^, we made efforts during BH15 towards semantic standardization of orthology content ^33^.

We developed the Orthology Ontology (ORTH ^34^, and 35) to capture essential concepts pertaining to orthology, including clusters of orthologs derived from speciation events. ORTH was designed following best practices in ontology engineering, i.e., reusing related ontologies such as the Semanticscience Integrated Ontology (SIO, ^36^), the Relations Ontology (RO, ^37^), and the Comparative Data Analysis Ontology (CDAO, ^38^). We used the Semantic Web Integration Tool (SWIT, ^39^ and ^40^), a generic tool for generating semantic repositories from relational databases and XML sources, to convert InParanoid, OMA, and TreeFam datasets in OrthoXML format into RDF. More details and sample queries for the datasets using ORTH are on the source code repository ^41, 42^.

Although the standard mapping and transformation by SWIT was largely able to transform the content of the three databases, though a few resource-specific rules were necessary because: (1) OrthoXML offers generic tags that are used by orthology databases in a heterogeneous way, e.g. for describing the taxonomic range of a cluster of orthologs; and (2) different orthology resources use identifiers of genes or proteins from different databases, so the corresponding prefixes for URIs had to be adapted. The next steps include: (1) evaluation of the results by the Quest for Orthologs (QfO, ^43^) community, which could lead to the development of a QfO semantic infrastructure for sharing orthology resources; (2) examining the interoperability of semantic orthology datasets using additional databases such as UniProt ^44^, KEGG OC ^45^, and the Microbial Genome Database (MBGD, ^46^); and (3) developing applications and tools for comparative analysis of genomes/proteomes utilizing the ORTH.

***Molecular evolutionary process calibration.*** Not only qualitative but also quantitative representation of evolutionary events, i.e. on a time axis, among organisms is important for evolutionary biology. However, the adoption of Semantic Web technologies is lagging behind in domains of the biological sciences outside of the conventional scope of BH15. For example, in recent years several hackathons and other meetings have been held to address challenges of data mobilization ^47^ and integration in phyloinformatics ^48, 49^ and biodiversity informatics ^16^ that uncovered a paucity of web services that deliver ontologized, or even machine-readable, data on fossil specimens. Although expected waiting times between speciation events can be modeled ^50^, fossils are needed for calibrating phylogenies to absolute time axes ^49, 51, 52^, e.g. to detect nucleotide substitution rate shifts coinciding with evolutionary events such as speciations, which generate orthology, and gene duplications, which generate paralogy.

Recently, a working group at the National Evolutionary Synthesis Center (NESCent, ^53^) initiated a project to address this ^54^ and to establish a database of reference fossils with a web service API ^55^. To evaluate whether this new resource can indeed be usefully applied in the analysis of molecular data we developed a proof-of-concept pipeline ^55^ (based on Bio::Phylo ^56^ and SUPERSMART ^57^) that includes a reconciliation between fossil taxa from the FossilCalibrations database and extant taxa from in the TreeFam orthology database. The steps are as follows:

1. Download a data dump release from TreeFam.
2. For each TreeFam gene family, fetch fossils from FossilCalibrations through the API. This was done by querying for the taxonomic names, e.g. “Mammalia”, that are applied to internal node labels in gene family trees.
3. Apply the fossil ages as calibration points for a penalized likelihood analysis using *r8s* ^58^.
4. Using the produced ‘ratogram’ (a phylogenetic tree whose branch lengths are proportional to inferred substitution rates, one of the results produced by the *r8s* analysis), calculate the substitution rate as a function of time since the most recent gene duplication event.

**Figure 2.**
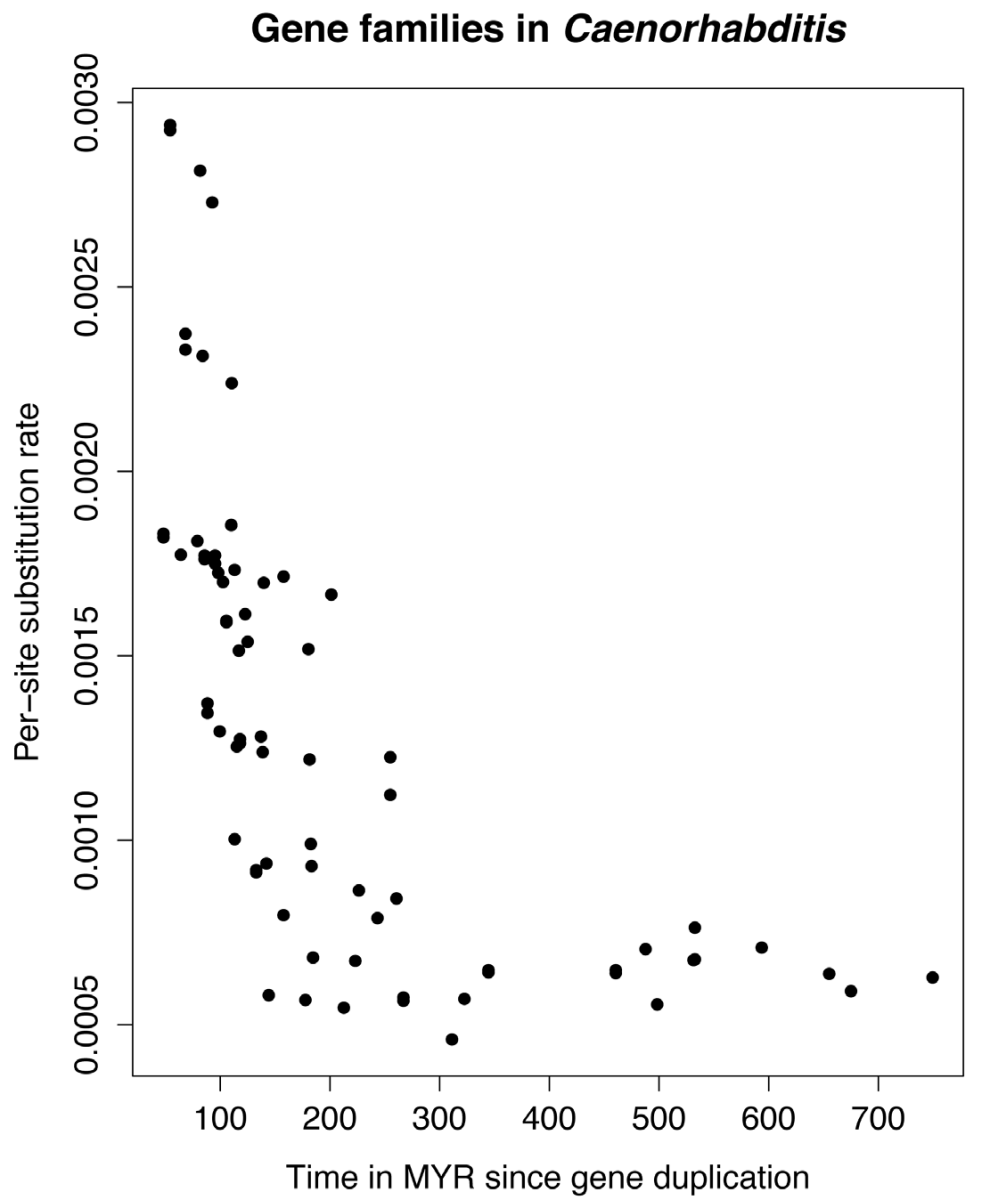
Substitution rates as a function of evolutionary distance since the age of the most recent gene duplication observed in *Caenorhabditis* genomes.

The rationale for this pipeline was that the general model of gene duplication followed by neo- or subfunctionalization ^59^ suggests that reconstructed substitution rates (which are retrospective, and based on accumulated fixed mutations) should be elevated in novel gene copies that are either under relaxed or under directional selective pressure. Hence, we would expect to see elevated substitution rates following a duplication event, which should taper off over time. Given that baseline substitution rates differ between lineages we performed an assessment of whether this prediction could be detected confined to a single lineage, that of *C. elegans.* Figure 2 suggests that this is indeed the case (this is in essence a different way of obtaining, roughly, some of the findings of ^60^). As a proof of concept to test whether it is possible to include fossil data from this new resource we conclude that this is indeed possible, but we note several drawbacks:

- The FossilCalibrations database makes its data available as simple JSON. This is convenient for programmers but it also means that certain concepts used in the JSON are ambiguous as they are not linked to any controlled vocabulary or ontology.
- The distinction between stem and crown fossils is made using magic numbers whose values and their meanings are poorly documented (we could only discover their semantics by inspecting the source code of FossilCalibrations).
- Some of the taxon names used by FossilCalibrations are not scientific names from any explicitly identified taxonomy. For example, some fossil calibration points have names such as “Chimpanzee-Human”, or “Humanity”. Such names are difficult to resolve using taxonomic name resolution services.
- There are large biases in taxon sampling in the database. In fossil databases this is nearly inevitable as some taxa fossilize much better than others, but even where a relatively rich fossil record is known to exist, e.g. in the sea urchins (Takeshi Kawashima, pers. comm.), no records were available in the database.

The first three drawbacks we identified can all be traced back to poorly defined semantics, which we therefore characterize as the key current issue in LOD representation of fossil specimens. To fill this gap, firstly we need to semantically curate FossilCalibrations data manually, which of course may take time, then export curated information in RDF so that analyses proposed in this section can be integrated in the automated pipeline.

### Proteomics

***Protein semantic representation.*** Many datasets on the Semantic Web are available as RDF, but often lack the explicit model-theoretic semantics provided by languages such as OWL. For complex datasets, the additional semantics of OWL, which includes assertions of disjointness, i.e. the explicit semantic distinction between classes and their instances, and axioms restricting the use of classes and object properties, may be particularly beneficial. The main limitation of languages such as OWL is that querying them is often highly computationally intensive and therefore not feasible for large datasets. Our aim was to evaluate how well formal languages like OWL scale in representing very large datasets. We chose the UniProt database ^44^, as it currently constitutes one of the larger RDF datasets, is used throughout biology, and has rigorous quality checks. Our aim was to find a representation of proteins and their functions using OWL. As automated reasoning over OWL knowledge bases is highly complex (2-NEXPTIME complete), we limited ourselves to the OWL 2 EL profile. However, widely used ontology design patterns for representing functions are not expressible in OWL 2 EL, as certain types of restrictions (in particular universal quantification) do not fall within the OWL 2 EL expressivity. As a consequence of these limitations, we decided to develop a novel representation pattern for asserting that proteins have a function that would fall in OWL 2 EL and would enable us to convert all of UniProtKB into OWL (though for testing purposes we converted only a subset on the order of 10 ^5^ OWL axioms). Specifically, given proteins XYZ, we generate the following classes:

- Class XYZ (instances of this class are individual proteins)
- Class XYZ_all (instances are the sets of all XYZ proteins in the universe; intuitively, only one instance of this class can ever exist)
- Class XYZ_isoform for all isoforms of XYZ
- Class XYZ_generic (the 'generic' form of the protein, i.e., a group of orthologous proteins)
- We also generate the following axioms (here expressed in Manchester OWL Syntax):
- XYZ SubClassOf: XYZ_generic
- XYZ_isoform SubClassOf: XYZ
- XYZ_isoform SubClassOf: isoform-of some XYZ
- XYZ SubClassOf: member-of some XYZ_all
- XYZ_all SubClassOf: { xyz } i.e., XYZ_all is a singleton class, and lower-case xyz is a new constant symbol that is newly introduced for each axiom of that type
- XYZ_all SubClassOf: has-member only XYZ (XYZ_all is homogenic)

Of these axioms, only the last axiom (XYZ_all is homogenic) is not expressible in OWL 2 EL, while all other axioms can be expressed in the OWL 2 EL profile. We have converted several types of proteins from UniProtKB using this approach and evaluated queries and query time. However, a thorough analysis on how well this approach scales to ontologies of the size of UniProtKB is left for future work. The source code developed for this project is available at our source code repository ^61, 62^.

***Proteome assay annotation.*** In proteomics, expressed proteins are usually identified by mass spectrometry. In most common workflows, proteins are digested into peptides with a protease. The peptides are ionized and then fragmented. Their precursor mass-to-charge ratios and fragment ion spectra are experimentally measured and compared with theoretical masses and fragmentation patterns of peptides calculated from a protein database. Information about experimental protocols and data analysis methods is thus important for understanding the raw and processed data. An identified protein list has substantial amounts of metadata such as labels used for quantification, e.g. iTRAQ, ^63^, or SILAC, ^64^, protease used for protein digestion (most commonly trypsin), pre-separation method (LC, 2D-gel electrophoresis, etc.), ionization and ion detection method of the mass spectrometer (MALDI-TOF-TOF, etc.), peak-processing software (ProteoWizard, ^65^; MaxQuant, ^66^; etc.), protein database used for theoretical peptide mass calculation (UniProt, ^44^; Ensembl, ^67^; etc.), database search software for peptide-spectral matches (Mascot, ^68^; X!Tandem, ^69^; MaxQuant, ^66^; etc.), and parameters and thresholds of the software. These experimental protocol- and data analysis method-related terms are necessary metadata for submissions to proteome databases/repositories.

To describe these metadata, the Human Proteome Organization Proteomics Standards Initiative (HUPO-PSI) has developed the PSI-MS controlled vocabulary ^70^ and ProteomeXchange ^71^, which is a consortium of mass spectrometry proteomics data repositories including PRIDE, the Peptide Atlas SRM Experiment Library (PASSEL, ^72^), and MassIVE ^73^, has established a core set of metadata for dataset deposition using PSI-MS.

The Japanese proteome community is now developing the Japan Proteome Standard (jPOST) repository ^74^, which is a mass spectrometry proteomics data repository. The salient feature of jPOST is the ability to re-analyze data from deposited raw data; by using raw data and a jPOST-original re-analysis workflow, the community plans to integrate data from various experiments to construct a standardized proteome database (jPOST database). Original analytical results from submitters are not suitable for integration because they were performed using various different protein databases and peak-identification/database search software with various different parameters.

**Figure 3.**
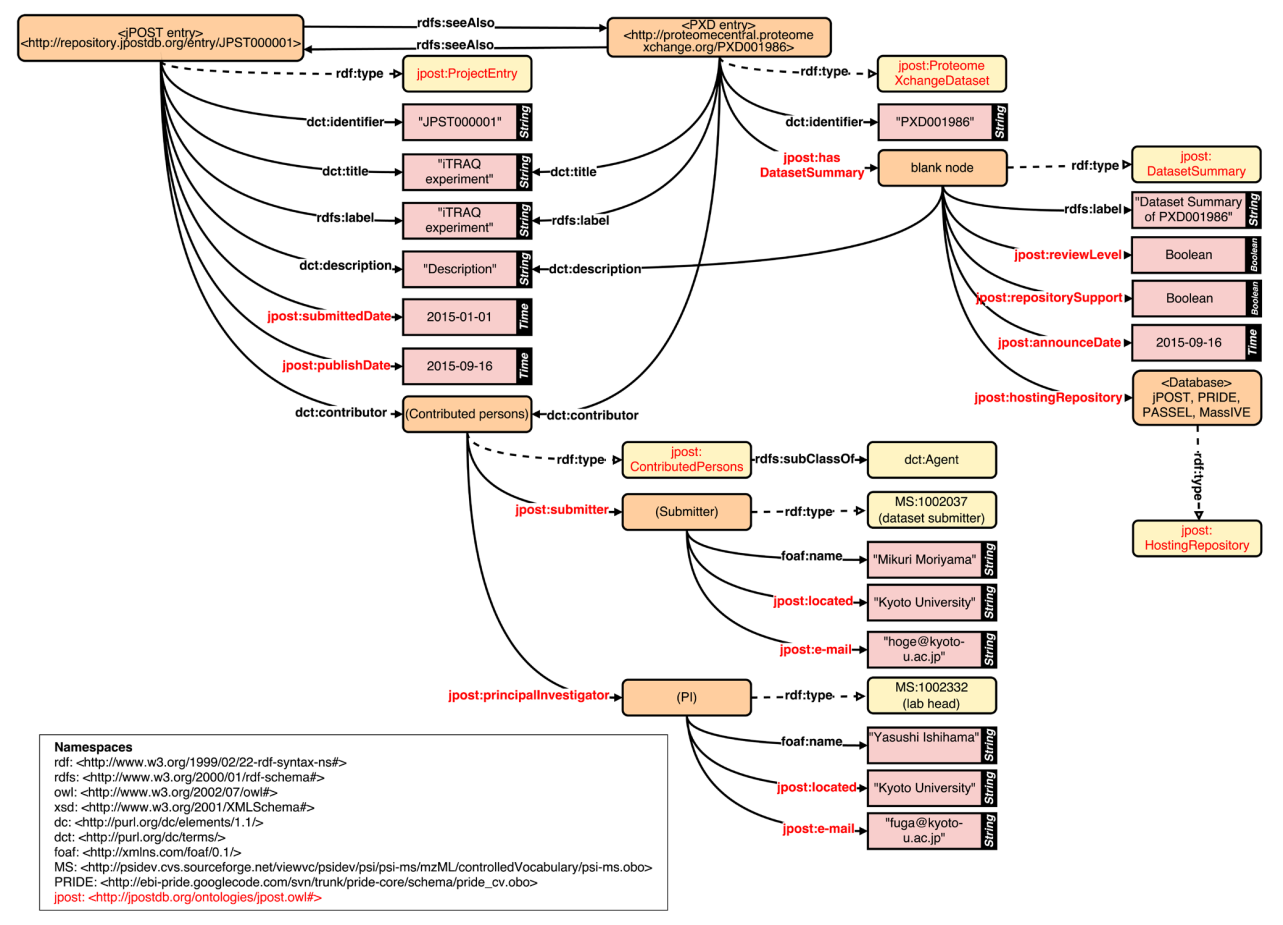
RDF schema for jPOST metadata.

For re-analysis, it is necessary to describe detailed information about experimental procedures. However, current controlled vocabularies (CVs) such as PSI-MS are insufficient for metadata description, and so we have attempted to reorganize and extend the current CVs for jPOST. At BH15, we enumerated required categories of metadata, such as Instrument mode and Quantification platform, and collected vocabularies with the cooperation of experimental proteomics scientists. The collected vocabularies were mapped to existing CVs where possible, and we began to develop an ontology for unmapped vocabularies ^75^. We also developed an RDF schema based on the CVs and ontology ( Figure 3) for jPOST datasets. Constructing an ontology that is compatible with existing CVs such as PSI-MS is important for integrating jPOST data with other proteomics data stored in the databases of the ProteomeXchange Consortium ^71^. In addition, by using common ontologies/CVs such as Taxonomy and disease name and a standardized data model like RDF, the proteomics datasets can also be linked and integrated with datasets derived from other technologies such as transcriptomics and epigenomics.

### Metabolomics

***Tools for metabolite identification and interpretation.*** Metabolomics is the biochemical analysis of all low-molecular-weight metabolites in a biological system, i.e. the metabolome. Owing to the chemical diversity and complexity of the metabolome, no single analytical platform can detect all metabolites in a sample simultaneously. Current state-of-the-art approaches for measuring metabolites and maximizing metabolite coverage require integration of multiple analytical platforms, data pre-processing methods, effective metabolite annotation, and data interpretation ^76, 77^. Given that the most commonly used analytical and data pre-processing methods have been comprehensively reviewed ^78– 80^, we will not discuss them here, but rather focus on downstream analyses such as pathway analysis, and effective data interpretation.

Scientists in natural products chemistry use the accurate mass and chemical shifts in Nuclear Magnetic Resonance (NMR) spectra to elucidate the structure of unknown natural chemical compounds. In contrast, researchers in metabolomics commonly try to provisionally identify chromatographic peaks by comparing their retention time (or retention indices), and/or the mass spectra, with those present in a mass spectral library database generated from the data of authentic standards ^81^. The Metabolomics Standards Initiative defined four levels of reporting metabolite identification and annotation: identified metabolite (Level 1), putatively annotated metabolites (Level 2), putatively annotated metabolite classes (Level 3), and unknown compounds (Level 4) ^82^. This indicates that the confidence levels of metabolite identification reported in metabolomics studies can vary largely, because of different extraction protocols, different instruments and measurement parameters, different pre-processing methods, and the diversity of annotation expertise ^83^. This hampers the reusability/reanalysis of published metabolomics datasets, although there are public repositories for metabolomics data such as MetaboLights ^84^ and MetabolomeExpress.org ^85^.

Biological interpretation of changes in metabolite levels is very important and is still challenging, because such metabolite pools are the resulting output of many biological processes. To facilitate biological interpretation by existing biological knowledge, e.g. biochemical pathways, pathway-based analysis like Metabolite Set Enrichment Analysis (MSEA, ^86^) is available. This approach highly depends on predefined biological pathways such as KEGG ^87^, Pathway Commons ^88^, BioCyc ^89^, and WikiPathways ^90^. Molecular interactions can be regarded as a network by calculating association between molecules in omics data. Correlation-based approaches are behind for construction of association networks such as gene co-expression networks in transcriptomics (for example, see 91, 92).

There are many software tools for pathway visualization and integration of different omics data (for example, see 93). Examples include KEGG Mapper ^94^, KEGGViewer ^95^, PathVisio ^96^, WikiPathways App ^97^, and KEGGScape ^98^. Metscape is a Cytoscape App for network analysis and visualization of gene-metabolite associations ^99^. MetaMapR ^100^ can be used for integrating biochemical reaction with chemical structural and mass spectral similarity to analyze pathway-independent associations including unknown metabolites. MetaboAnalyst ^101^ provides a user-friendly, web-based analytical platform for metabolome data pre-processing, normalization, statistical analysis, and metabolite annotation. DeviumWeb ^102^ is also a user-friendly web application for integrating statistical multivariate analysis with biochemical domain knowledge using R-Shiny ^103^, a web application framework for R.

***Plant metabolome database development.*** Unlike compound and mass spectral databases such as KEGG ^87^ and MassBank ^104^, metabolite-profile oriented databases still remain relatively undeveloped and under-used in plants ^81^. The data and metadata for more than 140 mutants of *Arabidopsis thaliana*, an important model plant, are archived at the Plant and Microbial Metabolomics Resource (PMR, ^105^). It is a flexible database that is designed for data sharing in metabolomics and implements data analysis tools ^106^. Information on phenotypic screening of *Arabidopsis* chloroplast mutants using assays of amino acids and fatty acids of more than 10,000 T-DNA insertion mutants using mass spectrometry are stored in Chloroplast 2010 ^107– 109^.

We recently developed a new database, the Metabolite profiling database for Knock-Out mutants in *Arabidopsis* (MeKO, ^110^), to facilitate improvement of gene annotation. The MeKO database ^111^ can be used to browse and visualize metabolomic data, containing images of mutants, data on differences in metabolite levels, and the results of statistical data analyses. As mentioned above, the metabolomics community is working towards the setup of sharing metabolome data, while mining publicly available information and demonstrating the richness of integration of multiple metabolome datasets that remain largely unexplored. At present we are constructing our database, called AtMetExpress ^112^, to store this information. It is freely available and contains detailed information about metabolites detected in *Arabidopsis*. It has a small and simple GUI tool for performing meta-analyses, allowing easy metabolome meta-analysis for plant biologists using R-Shiny.

Plants produce a diversity of compounds through secondary metabolic pathways. In these secondary compounds, the flavonoids and glucosinolates are useful as herbal medicines to maintain human health. However, a lot of them are still undescribed in public pathway databases. It is therefore important to construct the infrastructure to integrate such metabolites with their pathways in a cross-database manner. Hence, compounds IDs need to be linked rationally for this purpose.

To address the above challenge, we focused on the following things at BH15. We tried to implement several web applications with R-Shiny to improve visualization tools in our metabolome database, AtMetExpress. To reconstruct secondary metabolite pathway maps on WikiPathways we curated metabolite name, database identifiers of metabolites and reactions (KEGG, KNApSAcK, PlantCyc, and PubChem) in *Arabidopsis* metabolome data. We focused on flavonoids, which is a well-studied secondary metabolite group in *Arabidopsis*. We developed the following web applications and tools: a webapp called the Prime Visualization Tool using the R-Shiny framework; an integrated “pathview” Bioconductor package with the Prime Visualization Tool ^113^; an R package for the linkdb RDF client ^114^ to integrate multiple identifiers of major compound databases like PubChem CID, KEGG, and KNApSAcK.

In addition, we examined the SummarizedExperiment container ^115^ in Bioconductor to use assay, and we discussed the possibility of using the SummarizedExperiment in RDF format. We integrated several *Arabidopsis* metabolome datasets and partly finished data curations. These curation efforts continue after BH15. Even in the model plant *Arabidopsis*, the main target of existing large-scale metabolic models was primary metabolism (for example, see 116– 119). Our effort to construct curated *Arabidopsis* flavonoid dataset will help to expand metabolic models of *Arabidopsis* and lead to a better understanding of the production of flavonoids.

### Biochemical molecules

***Chemical database integration.*** Small molecules are studied across a broad set of research areas. They are important as a vital component of living systems and are also used in the formulation of pharmaceutical products. Therefore, access to information collected about molecules is key to research and product development. During BH15, we discussed strategies for cooperation between chemical databases. For instance, participants discussed the role of InChIKey ^120^ in their own databases as a primary key for chemical structure. Other discussions focused on increasing interoperability in two ways: First by including additional database cross-references, and second by harmonizing the RDF representation of chemical data. Chemical databases such as PubChem ^121^, Nikkaji ^122^, GlyTouCan ^123^, and the Protein Database Japan (PDBj, ^124^) store data in atomic level formats such as Molfile ^125^, mmCIF ^126^, InChI ^120^, and InChIKey. Participants agreed to use ontologies such as SIO, the Chemical Information ontology (CHEMINF, ^53^), and the Simple Knowledge Organization System (SKOS, ^127^). The RDF data of Nikkaji, KNApSAcK ^128^ and GlyTouCan were modified to use these ontologies. Increased adoption of the ontology-based RDF representation of small molecules will facilitate their integration and reduce the cost of reuse of data from each of the databases.

***Chemical transformation annotation.*** We previously developed an ontology for annotating biochemical transformations called Partial Information of chemical transformation (PIERO, ^129^). PIERO provides vocabulary to describe transformations and their attributes along with sets of possible reactions. The vocabulary enables the examination of similar enzymatic reactions, which is particularly important for reactions for which no enzyme has been identified yet. Such reactions are common in secondary metabolism found only in limited organisms. In most cases, they are just putative substrate-product relationships and the reaction equations are not characterized completely. During BH15, we augmented PIERO in a number of ways, including improved RDF interoperability, data curation (adding/correcting more terminology), and reviewing the classification criteria for transformations. One of the most important developments was in the definition of a classification based on reaction characteristics, including the gain or loss of groups, opening or closing the ring structures, intermolecular transfer of groups, formation/digestion of groups, transfer/exchange of groups, and the steps of the reactions.

***Glycomics ontology development.*** Carbohydrates, often referred to as glycans, differ from other biopolymers such as proteins or nucleic acids in the large variety of different building blocks, i.e., monosaccharides, and in the possibility of linking these building blocks in several ways, which often results in branched structures. Furthermore, experimental techniques for glycan identification often yield underdetermined structures with varying degrees of uncertainties. Many providers of glycoinformatics databases and tools have developed individual and non-compatible formats to store all these properties of glycan structures, such as LINUCS ^130^, LinearCode® ^131^, KCF ^132^, GLYDE ^133^, GlycoCT ^134^, or WURCS ^135^. This variety of nomenclature formats is a major reason for a lack of interoperability and data exchange between various glycoinformatics resources ^136, 137^. To overcome this situation, development of the glycomics standard ontology (GlycoRDF, ^138, 139^) was started during BioHackathon 2012 ^4^.

GlycoRDF can represent glycan structure information together with literature references or experimental data. MonosaccharideDB ^140^ provides GlycoRDF descriptions of monosaccharides generated from various carbohydrate nomenclature formats. During BH15, participants developed routines to generate GlycoRDF data from WURCS 2.0 nomenclature, which is used by the GlyTouCan structure repository ^123^. Thus, glycomics data can now be retrieved as GlycoRDF from GlyTouCan, GlycoEpitope ^75^, GlycoNAVI ^141^ and WURCS using database guidelines ^142^.

The group also discussed possible extensions to GlycoRDF that would offer relations between individual monosaccharides. Lactose, for example, is a disaccharide composed of β-D-galactopyranose (1-4)-linked to D-glucose. The latter can be of any ring form or anomeric state due to mutarotation. With relations such as “β-D-glucopyranose is_a D-glucose” or “α-D-glucofuranose is_a D-glucose”, the definition of lactose given above can be used to identify disaccharides with β-D-galactopyranose (1-4)-linked to β-D-glucopyranose or to α-D-glucofuranose as lactose as well. Options to derive such relations from WURCS 2.0 nomenclature have also been discussed. The encoding of these relations in RDF uses existing chemistry definitions such as SIO as much as possible. A first implementation of creating such relations automatically has been added to MonosaccharideDB. The resulting representation will enable (sub-)structure searches with different levels of information in query and target structures, and will also help to assign relations between oligosaccharides.

Glycoinformatics is at the intersection of bioinformatics and chemoinformatics. In the past there have mainly been attempts to establish cross-links between glycan databases and bioinformatics resources, e.g. between UniCarbKB ^143^ and UniProtKB ^44^, which makes sense from the point of view of glycoproteins and protein-carbohydrate complexes. From the small molecules perspective it is coherent to also cross-link with chemoinformatics databases such as PubChem ^121^ or Nikkaji (now subsumed by J-Global, ^122^). Glycan structures cooperation was discussed at BH15. As part of this process several possible formats for data exchange were discussed, such as SMILES ^144^, InChI, mmCIF, WURCS, or mol file. A focus was subsequently put on the conversion of glycan structures to SMILES, and routines to generate SMILES codes from monosaccharide names were developed in a cooperation between PubChem and MonosaccharideDB developers. This will provide an important bridge between glycoinformatics and chemoinformatics and will make it easier for people outside the glycoscience community to access glycomics data. For cooperation between GlyTouCan and PubChem, RDF triples were developed with GlycoRDF, SIO, CHEMINF, DCT, and SKOS.

The large variety of monosaccharides is mainly caused by the fact that the basic building blocks such as glucose or galactose are often modified by substituents that replace hydrogen atoms or hydroxyl groups, or by introduction of double bonds, deoxy modifications, etc. Currently, no explicit rules exist to define how many modifications can be made to a standard monosaccharide so that it can still be considered as a monosaccharide. Some possible criteria for discrimination between carbohydrate and non-carbohydrate residues were discussed at BH15. We developed a new approach for detecting carbohydrate candidate backbone skeleton. An algorithm for automatic detection of candidate carbon chains of monosaccharide was discussed.

## Research methods

### Data retrieval and querying

***OpenLifeData to SADI deployment.*** The Bio2RDF project ^145^ is now well known within the life sciences LOD community. Recently, OpenLifeData ^146^ completed an effort to provide a distinct view over the Bio2RDF data, with deeper and more rigorous attention to the semantics of the graph, and these views were provided through a distinct set of SPARQL endpoints, with each endpoint acting as a query-rewriter over the original Bio2RDF data ^147^. With these richer and more uniform semantics, it became possible to index each endpoint and automate the construction of SADI Semantic Web Services ^148^ providing discoverable, service-oriented access to all OpenLifeData/Bio2RDF data ^149^—a project that was named OpenLifeData2SADI.

Prior to BH15, the OpenLifeData endpoints were further consolidated into a single endpoint, which caused the OpenLifeData2SADI services to fail. At BH15, the SADI and OpenLifeData project leaders took the opportunity to rewrite the OpenLifeData2SADI automated service deployment codebase. This was originally written as an interdependent mix of Java and Perl scripts, which often took several days to complete. The new codebase is entirely Perl-based, and with the exception of the OpenLifeData indexing step, which is highly dependent on the size of the available OpenLifeData endpoints, runs in less than one hour, deploying tens of thousands of SADI Semantic Web Services over the refactored data. The speed of this new code makes it reasonable to rerun the service deployment dynamically as the underlying OpenLifeData expands or changes, or perhaps automate the re-deployment of services on, for example, a nightly basis. In an ongoing activity since BH15, re-indexing of OpenLifeData has made it possible to capture sample inputs and outputs for each of the resulting SADI services. This information will be added to the SADI service definition documents, allowing for automated service testing and/or more intuitive service registry browser design with, for example, pre-populated “try it now” functionality.

***SPARQL query construction.*** SPARQL ^150^ has emerged as the most widely used query language for RDF datasets. RDF datasets are often provided with web interfaces, called SPARQL endpoints, through which SPARQL queries can be submitted. However, constructing a SPARQL query is a relatively complex task for inexperienced users. SPARQL Builder ^151^ is a web application that assists users in writing SPARQL queries through a graphical user interface. The SPARQL Builder system interactively generates a SPARQL query based on a user-specified path across class-class relationships. At BH15, we worked on the display of candidate paths from metadata, including hierarchical information of the SPARQL endpoint, graphs, classes, properties, class-class relationships, and their statistics, such as the numbers of triples and instances. To be time efficient, we found that it was necessary to pre-compute and store those metadata for fast retrieval. This suggests that it would be ideal that every SPARQL endpoint provides such metadata. We tested our system on datasets drawn from the EBI RDF Platform and Bio2RDF, and our approach could be extended to other RDF datasets. We also developed a prototype ^152^ of a search interface using SPARQL Builder system for 439 datasets contained in the Life Science Database Archive (LSDB Archive, ^153^). The LSDB Archive is a service to collect, preserve and provide databases generated by life sciences researchers in Japan. Using the interface, we can now search for data in the LSDB Archive without knowing the data schema for each dataset.

***LODQA integration with DisGeNET and Bio2RDF.*** LODQA ^154^ is another service being developed to provide a natural language interface to SPARQL endpoints. Users can begin their search with a natural language query, e.g. *What genes are associated with Alzheimer’s disease?*, from which the system automatically generates corresponding SPARQL queries. LODQA also features a graph editor that allows users to compose queries in a graph representation. While the system is developed to be highly adaptable to any RDF datasets, it does require lexical terms, e.g. labels, of data sets to be pre-indexed.

During BH15, we explored the use of the LODQA system with DisGeNET and Bio2RDF. As a result, we found that LODQA could generate effective SPARQL queries for some natural language questions like "Which genes are involved in calcium binding?" The LODQA interface to Bio2RDF is publicly available ^155^, while the LODQA interface to DisGeNET is discontinued due to major revisions to DisGeNET.

***Crick-Chan query parsing.*** While LOD and the Semantic Web are rapidly adopted in the biology domain, the majority of biological knowledge is still only available in the form of natural language text, for example in manuscripts on PubMed or in textbooks on the NCBI Bookshelf. The ability to make use of this ocean of data would facilitate knowledge discovery and help bridge the current data retrieval process and the Semantic Web. The success of IBM Watson in the quiz show Jeopardy highlighted the potential of state-of-the-art cognitive computing in answering natural language questions. IBM Watson, however, does not rely so much on semantics or machine learning, but is rather based on queries on unstructured data, with statistical identification of answer domains (Lexical Answer Type). The software for IBM Watson (DeepQA) uses a system to answer a “word” that matches the natural language query by searching through millions of pages of documents, including the entire text of Wikipedia. A scientific fact, or indeed any knowledge, is almost always written in natural language in the form of a manuscript, use of which is relatively less explored in the Semantic Web context. Therefore, at BH15 the G-language Project team aimed to develop a software system, designated “Crick-chan”, that mimics DeepQA to find the most relevant “sentence” (as opposed to a “word” in Watson) from millions of scientific documents. Crick-chan mimics the architecture, and works as follows:

1. The question text first undergoes morphological analysis using Enju ^156^ to extract objective nouns and key verbs. Using a dictionary search, proper nouns are identified.
2. Queries are extended using the Bing search engine (which allows for the largest number of free queries among search engines). At the same time, the question is checked to see whether it belongs to the biology domain.
3. Full text searches are performed for the entire OMIM, PubMed, PubMedCentral, NCBI Bookshelf, Wikipedia, and the entire WWW, via queries to NCBI EUtils and Bing searches.
4. Relevant sentences are extracted from the most relevant matches.
5. Extracted sentences, i.e. the answer hypothesis, are checked for grammatical completeness and are scored according to keywords.
6. Answer confidence is scored according to the data sources and the completeness of key terms.
7. The resulting "answer" is presented in a user interface with an artificial character to assist the natural language query process.

**Figure 4.**
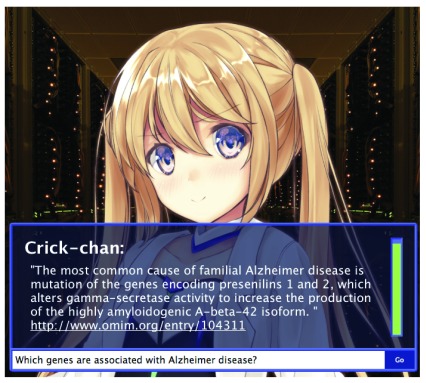
The graphical interface of Crick-chan as it answers which genes are associated with Alzheimer’s disease.

For other general conversation, Crick-chan embeds the AIML bot (ProgramV 0.09) for cases when the question is not considered to belong to the biology domain, and for when there are fewer than two keywords. Crick-chan is publicly accessible ^157^ and it can answer natural language questions such as “What genes are associated with Alzheimer disease?” ( Figure 4).

### Natural language processing

***Clinical phenotype text mining.*** Clinical phenotypes, i.e. symptoms and signs, are key for diagnosis and treatment decision-making, particularly for rare or complex disorders ^158^. Delayed or inaccurate diagnosis incurs high economic costs in addition to heavy psychological burden on patients and their families. Deep clinical phenotyping in combination with genotyping are increasingly seen as important components of a vision for precision medicine ^159^. However, vast amounts of phenotypic data available from social media, EHR, biomedical databases, and the scientific literature, are largely inaccessible to direct computation because they are solely available in a narrative form.

Natural Language Processing (NLP) involves the automatic extraction of relevant information from unstructured text and represents it in the form of structured concepts and relationships amenable to further computational analysis. The acquisition of phenotype data is particularly challenging due to the complexity of textual descriptions. Several efforts have explored the extraction of phenotypes from text. For example ^160^, assessed the contribution of feature spaces and training data size on support vector machine model performance for mining phenotypic information on obesity, atherosclerotic cardiovascular disease, hyperlipidemia, hypertension, and diabetes from clinical documents. In the domain of congestive heart failure ^161^, developed automated methods for extracting phenotypic information from clinical documents and from published literature. With the goal of matching phenotypic findings to their correlated anatomical locations as described in clinical discharge summaries ^162^, developed a named entity recognition method based on the Epilepsy and Seizure Ontology (EpSO, ^163^). In fact, a review of studies describing systems or reporting techniques developed for identifying cohorts of patients with specific phenotypes found that 46 out of 97 papers on this topic used techniques based on natural language processing ^164^. In addition, several phenotype-annotated datasets have been recently extracted from journal articles and EHR by using BioNLP and text mining methodologies ^158, 165– 169^.

The large-scale acquisition of phenotypic relationships from the literature enable a more complete view on the current knowledge, and thus, more efficient science. The use of text-mined data, i.e. information that is programmatically processed, aggregated and mined, shows much promise for some current challenges such as phenotype definition, hypothesis generation for research, understanding disease and pharmacovigilance. Therefore, their representation as linked data using Semantic Web and LOD approaches and the linking of the annotated literature with the linked data open new avenues for knowledge discovery to advance research and improve health care.

The curation of biomedical information extracted from scientific publications by text mining is an important current bottleneck for knowledge discovery of new and original solutions for a better health and quality of life. Manual approaches for data curation become more and more time demanding and costly, so that computer assistance in screening (document retrieval) and preparing data (information extraction) is unavoidable. Crowdsourcing approaches have been recently applied with high accuracy ^170^. Therefore, biocuration over the LOD will give a new opportunity to validate knowledge and adding evidence at the same time. The integration of curated and text mined data in the LOD opens new challenges for evidence and provenance tracking. Recent use of the nanopublication approach gives a mechanism for evidence, provenance and attribution tracking ^171, 172^. 

BH15 offered an opportunity to address different challenges related to the capture and analysis of human phenotype data. The text mining group focused its effort in the primary domains for deep phenotyping: acquisition of phenotype associations from journal articles, integration and alignment of annotation BioNLP tools, evaluation of secondary use of text mining corpora for knowledge discovery, semantic integration of text mined and curated data in the LOD, and curation of text mined data. All these tasks were pursued with a clear emphasis on standardization and interoperability between life sciences databases, text mined datasets and BioNLP tools, with the further aim to linking to the LOD.

***Natural language processing of drug effects and indications.*** Structured drug labels have been used as a source to collect rich representations of drug effects and indications ^173– 175^, and these text mined representations have been used in drug repurposing and identification of new targets for known drugs. The Side Effect Resource (SIDER, ^176^) contains a collection of text mined drug effects and indications, using the Unified Medical Language System (UMLS, ^177^) to represent the phenotypes. While the UMLS covers a wide range of clinical signs and symptoms, it does not cover the full set of phenotypes described in non-UMLS biomedical ontologies such as the human Disease Ontology (DO, ^178^) and the Mammalian Phenotype ontology (MP, ^179^).

During BH15, we developed an NLP pipeline that identifies the phenotypes occurring in structured drug labels. As vocabularies, we use the phenotype ontologies for mammals, in particular the the Human Phenotype Ontology (HPO, ^26, 166^), MP, and the DO. Furthermore, we also use the phenotypic quality ontology (PATO, ^180^), and the Foundational Model of Anatomy (FMA, ^181^), an ontology of human anatomy, as additional vocabularies. Text processing is performed using Lucene, which includes basic text normalization such as stop-word removal and normalization to singular forms. The resulting text-mined annotations of the structured drug labels are freely available ^182^. In the future, these annotations of drugs need to be further evaluated and integrated in linked datasets.

***Data analysis of text-mined corpora.*** The combination of high-throughput sequencing and deep clinical phenotyping offers improved capability in pinpointing the underlying genetic etiology of rare disorders. The accuracy of hybrid diagnosis systems is challenged by the vast number of associated variants, many of which lack phenotypic descriptions. At BH15, we sought to learn possible genotype-phenotype relationships from text mining. Specifically, we aimed to use text-mined corpora to learn associations between biological processes disrupted by gene mutations with externalized phenotypes. To do so, we combined two PubMed datasets: i) a dataset generated by the Biomedical Text Mining Group (BTMG) at NIH, comprised of automatically extracted named entities (MeSH terms, genes and mutations); and ii) a second one, generated by the Phenomics team at the Kinghorn Centre for Clinical Genomics (KCCG). The latter covered structured PubMed metadata, i.e., MeSH terms, keywords, etc., as well as HPO annotations. The consolidation of the two datasets, via common MeSH terms, resulted in a final corpus of 6.5M abstracts. To learn biological process – phenotype associations, we added biological process annotations from the Gene Ontology (GO, ^183^). Using the underlying diseases as latent variables (via MeSH terms) and summation as aggregation function, we produced an association matrix between 7,666 HPO terms and 10,438 GO Biological Process terms. The actual use of the matrix has been left for future experiments. Such experiments may cover various aggregation functions (e.g., instead of summation, to use a linear interpolation of the term frequency inverse document frequency (TF-IDF) values of the HPO terms) as well as its application to discovering dense networks of phenotypes – biological processes. The latter could be achieved via some of the following mechanisms:

- Hierarchical clustering and singular value decomposition (SVD) for ranking HPO - GO BP associations.
- Pre-clustering of HPO terms based on the HPO top-level abnormalities.
- Pre-clustering of GO BP terms using higher-level common ancestors.

***Integration of text-mined and curated disease-phenotype data.*** DisGeNET-RDF contributes to LOD with Gene-Disease Associations (GDAs) obtained from Medline by text mining and integration with associations from different authoritative sources in human genetics ^184^. From release 3.0.0, DisGeNET-RDF also integrates curated Disease-Phenotype Associations (DPAs) to HPO terms for diseases in OMIM, Orphanet, and DECIPHER ^185^ from the HPO project ^25^. In order to examine what are the challenges to integrate text mining with curated DPAs in LOD, we analyzed the DPAs in DisGeNET-RDF (v3.0.0) and the DPAs text-mined from the scientific literature by Hoehndorf *et al*. ^167^.

- *Hoehndorf2015*: This text-mining DPAs dataset contains 6,220 diseases identified by DO identifiers (DOIDs), 9,646 phenotypes identified using the HPO and the MP, and 124,213 DPAs.
- *HPO2015*: This curated DPAs dataset contains 113,203 DPAs between 7,841 diseases and 6,838 phenotypes from OMIM, Orphanet and DECIPHER data sources in which diseases are identified by the corresponding database identifier of provenance, and phenotypes are uniformly identified by HPO identifiers.

We normalized 6,220 diseases from the Hoehndorf2015 dataset to 5,194 UMLS CUIs by DOID-UMLS cross-references extracted from DO version 2015-06-04 with which only 75% (4,648) of DOIDs can be mapped to UMLS concepts. This is because 17% (1,088) of diseases are described with obsolete DOIDs and 8% (484 DOIDs) do not map to UMLS. Additionally, not all are 1:1 mappings, some N:1 DOID-CUI mappings exist. Therefore, phenotype annotations for different diseases will collapse in a unique UMLS concept.

**Figure 5.**
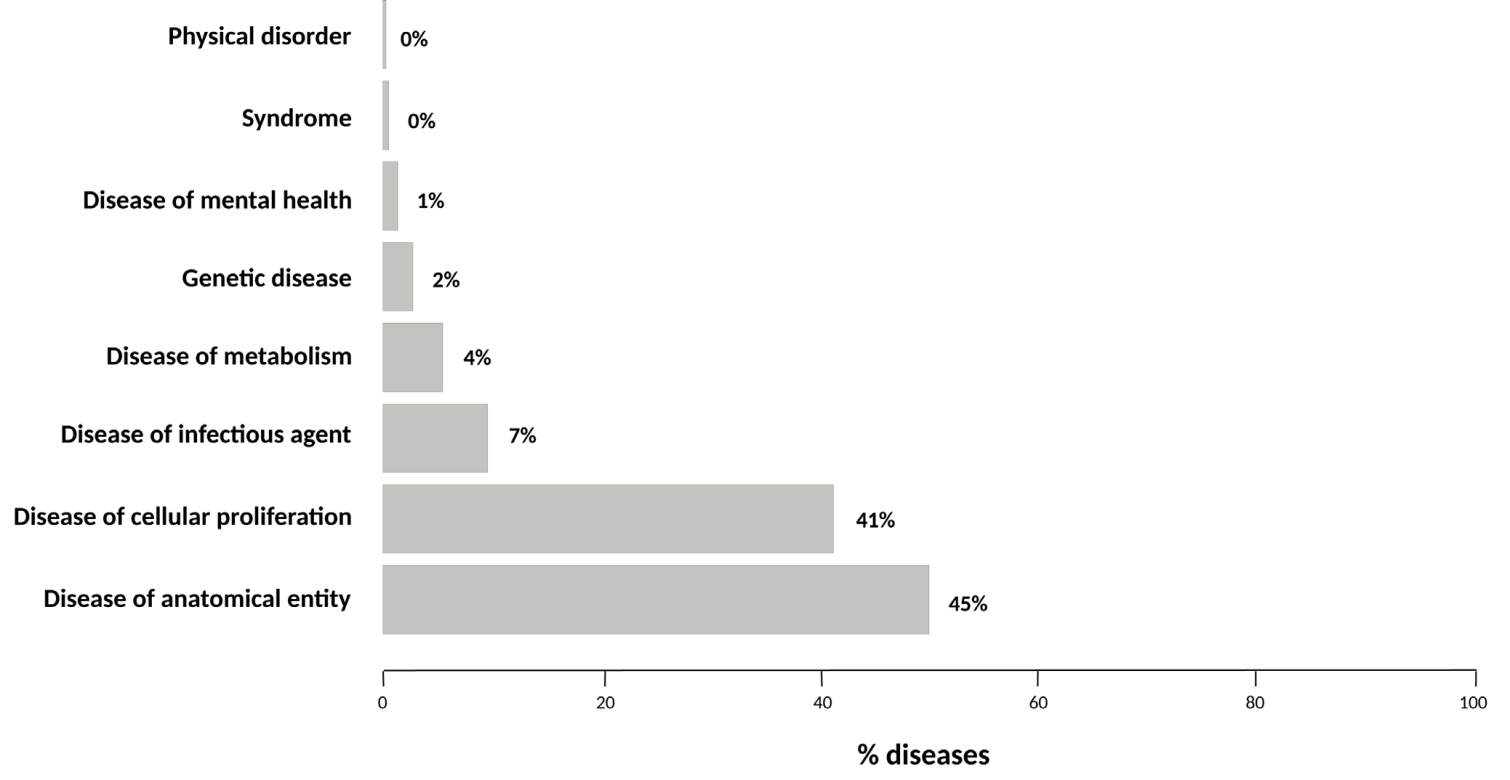
Disease coverage for top-level categories in DO of the diseases only annotated in the Hoehndorf2015 DPA dataset in comparison to the HPO2015 annotation.

The integration of the HPO2015 and Hoehndorf2015 datasets (9,067 and 5,194 UMLS CUIs, respectively) covers 13,596 UMLS concepts of the disease spectrum, of which only 3.2% (665 UMLS CUIs) are in both datasets. This low overlap is due to the fact that each project mainly focuses on covering different disease areas. Whilst the HPO annotation is intended to annotate Mendelian and rare genetic diseases, Hoehndorf *et al*.’s large-scale literature extraction was focused on broadening the disease class landscape to infectious, environmental, and common diseases. To characterize the disease coverage yielded only by text mining; in Figure 5 we show the top-level DO categories where these novel diseases fall. As can be seen, these novel findings mostly fall in ‘Disease of anatomical entity’ (DOID:7) and ‘disease of cellular proliferation’ (DOID:14566).

In summary, the analysis of aggregation and integration of text mined and curated disease-phenotype associations in DisGeNET highlights the potential value of text mining in data completeness, annotation, integration, and network biology, which can be used for instance for disease-phenotype ontology construction and curation, knowledge base population, and document annotation. The large-scale integration and publication of text mining DPAs in DisGeNET-RDF opens inference opportunities to grasp potential novel gene-phenotype associations from the current knowledge that promotes our understanding about disease etiology and drug action. However, it is important to keep track of machine-readable provenance and evidence at relationship level for computational analysis and credible knowledge discovery using LOD. Finally, the increase of disease/phenotype terminology and ontology mapping is crucial to foster semantic interoperability and data coverage.

***Assessing interoperability of disease terminologies.*** One benefit of improving the interoperability of disease terminologies is to facilitate translational research and biomedical discovery. Phenotype information is represented using terminologies, vocabularies, and ontologies, but the diverse phenotype spectrum poses serious challenges for their interoperability. For one, phenotypes span from the molecular to the organismal. In addition, while phenotypes in the biological domain are recorded as results from biological experiments, phenotypes in the clinical domain are used to report the state condition of patients ^186^. Furthermore, in current clinical nomenclatures for phenotypes such as MeSH, the 10th revision of the International Statistical Classification of Diseases and Related Health Problems (ICD-10), the nomenclature of the National Cancer Institute (NCI), SNOMED Clinical Terms (SNOMED CT), and UMLS, concepts are covered inconsistently and incompletely ^186^. All these issues affect ontology interoperability, and thus, the quality of their applications. The systematic ontological coding of phenotypic and molecular information in databases and their linking facilitates computational integrative approaches for identifying novel disease-related molecular information ^187^, prioritizing candidate genes for diseases ^188– 191^, as well as predicting novel drug-target interactions, drug targets, and indications ^192, 193^. The quality of the phenotypic descriptions of a resource will have implications for the quality of their interoperability, and thus, the quality of computational data analyses performed for translational research and knowledge discovery.

In DisGeNET-RDF (v3.0.0), diseases are normalized with the UMLS CUIs, and are mapped to several disease vocabularies/ontologies with different coverage (see 194 to see disease mapping coverage statistics). Much of the disease data in the data sources of the European Bioinformatics Institute (EBI) is annotated with EFO, such as BioSamples, which aggregates sample information for reference samples and samples used in multi-omics experiments, and the Gene Expression Atlas, which collects gene expression experiments. EFO disease terms have mappings to UMLS, DOID, MeSH, SNOMED CT, OMIM, HPO and ICD-10. EFO also includes and reuses terms from external terminologies such as disease/phenotype terms from the DO, the HPO, and rare disease terms from the Orphanet Rare Disease Ontology (ORDO, ^195^) that include some additional mapping to OMIM and UMLS. In this regard, during BH15, we aimed to increase the integration of DisGeNET and EBI data, by way of its RDF platform.

We assessed the coverage of EFO concepts against UMLS; from a total of 5,260 terms, only 52 map to the UMLS (see Table 3). Some disease terms do not have cross-references to UMLS concepts. For instance, cancer (EFO_0000311) does not have UMLS CUIs associated, even though it is a general disease term. Nevertheless, the EFO contains over 2000 UMLS mappings from other ontologies, most of them from ORDO, which are manually curated. We suggest that an increase in the mapping between EFO and the UMLS terminologies will benefit data integration and interoperability between RDF datasets such as DisGeNET and other databases that are part of EBI RDF platform.

***Semantic haiku generation.*** Natural language generation is the longstanding problem of generating textual output from textual or non-textual sources ^196– 200^. The field has a number of potential applications in the life sciences ^201– 205^. One of the projects of BH15 included the construction of a “semantic” haiku generator. Realizing the potential of language generation in communicating information both to scientists and to the public in a way that is acceptable to readers requires the ability to generate text that meets user expectations regarding discourse cohesiveness, genre-appropriate characteristics of word structure, e.g. length, and the like. Poetry generation has been an active area of research in computational linguistics and natural language processing for some time. Here we extend the task definition to the use of LOD, and to the haiku structure, which has not previously been treated in the language generation literature ^42, 206– 210^. A haiku is a type of poem traditional to Japan; it consists of three verses with five, seven, and five syllables. In light of the work on semantic resources, in particular RDF datasets available through SPARQL, the idea arose to generate a haiku from a SPARQL query by identifying a connected subgraph in which the labels of the resources, or the properties linking them, follow the 5-7-5 syllable pattern of a haiku. Using the CELEX2 dictionary ^211^, which maps English words to their syllables, we wrote a small haiku generator that can be initialized with a SPARQL endpoint and a start node (a resource) from which a search is started to identify a subgraph with the haiku pattern. The prototype code is available at our source code repository ^62, 212^. An initial test of the script using the UniProt SPARQL endpoint together with the human Amyloid beta ^213^ protein, which resulted in the following haiku:

*Amyloid beta*

*protein classified with blood*

*Coagulation*

To the best of our knowledge, this is the first “semantic” haiku. Although it follows the haiku pattern, additional work is still required to generate haikus that have additional haiku qualities, in particular the occurrence of a word related to one of the four seasons, as tradition requires.

### Reproducibility

***Extending the Common Workflow Language.*** Computational genomics faces challenges of scalability, reproducibility, and provenance tracking. Larger datasets, such as those produced by The Cancer Genome Atlas ^214^, are now petabyte-sized, while procedures for read mapping, variant calling, genome assembly, and downstream imputation have grown impressively sophisticated, involving numerous steps by various programs. In addition to the need for reproducible, reusable, and trustworthy data, there is also the question of capturing reproducible data analysis, i.e. the steps that happen after raw data retrieval. Genomics analyses involving DNA or RNA sequencing are being used not just for primary research, but now also within the clinic, adding a legal component that makes it essential that analyses can be precisely reproduced. We formed a working group on the challenges of creating pipelines for reproducible data analysis in the context of semantic technologies.

With the advent of large sequencing efforts, pipelines are getting wider attention in bioinformatics now that biologists regularly have to deal with terabytes of data ^215^. This data can no longer be easily analyzed on single workstations, requiring that analysis is executed on computer clusters and analysis steps are run both serially and in parallel on multiple machines, using numerous software programs. To describe such a complex setup, pipeline runners, or engines, are being developed. 

One key insight from this development is that versioned software is a form of data and can be represented with a unique hash value, e.g., a Secure Hash Algorithm (SHA) value can be calculated over the source code or the binary executables. Also, the steps in a pipeline can be captured in scripts or data and can be represented by a unique hash value, such as calculated by git. This means that the full data analysis can be captured in a single hash value that uniquely identifies a result with the used software and executed analysis steps, together with the raw data.

We worked on the Common Workflow Language (CWL, ^216^), which abstracts away the underlying platform and describes the workflow in a language that can be used on different computing platforms. To describe the deployed software and make reproducible software installation a reality we also worked on virtualization (Docker) and software packaging and discovery (GNU Guix).

The CWL is an initiative to describe command line tools and connect them together to create workflows. The original idea of CWL is that a workflow can be described in a ‘document’ and this workflow, once described, can be rerun in different environments. CWL has roots in “make” and similar tools that determine order of execution based on dependency graphs of tasks. Unlike “make”, CWL tasks are isolated and the user must be explicit about its inputs and outputs thereby creating a (hopefully reproducible) document of the workflow. The benefits of explicitness and isolation are flexibility, portability, and scalability: tools and workflows described with CWL can transparently leverage software deployment technologies, such as Docker, be used with CWL implementations from different vendors, and are well suited for describing large-scale workflows in cluster, cloud, and high-performance computing environments where tasks are scheduled in parallel across many nodes.

At BH15, CWL support was added for the Toil workflow engine ^217^ and work was done on Schema Salad, which is the module used to process YAML CWL files into JSON-LD linked data documents. A tutorial was given on the Common Workflow Language to interested participants. CWL also added the ability to pipe-in JSON objects containing the parameters necessary to run a CWL-wrapped tool ^218^. This allowed CWL to be more easily used with Node.js Streams and thus with the Bionode.io project.

***Docker container registry development.*** One challenge is the creation of standard mechanisms for running tools reproducibly and efficiently. Container solutions, such as Docker, have gained popularity as a solution to this problem. Container technologies have less overhead than full virtual machines (VMs) and are smaller in size. At BH15, we started a registry of bioinformatics Docker containers, which can be used from the CWL, for example. From this meeting evolved the GA4GH Tool Registry API ^219^ that provides ontology-based metadata describing inputs and outputs. Work was also done on an Ensembl API in Docker ^220^.

To facilitate access to triple stores, we developed a package called Bio-Virtuoso based on Docker. The virtuoso-goloso container runs an instance of the Virtuoso triple store ^221^. This container also receives Turtle, RDF/XML, and OWL format files via the HTTP Post method and internally put them into Virtuoso speedy using the *isql* command. Graph-feeding containers download data from sources, convert them into RDF if necessary, and send them to virtuoso-goloso. Multiple graph-feeding containers can be combined on demand. To date, we have supported data sources such as the HPO, HPO-annotation, Online Mendelian Inheritance in Man (OMIM, ^222^), OrphaNet ^223^, the HUGO Gene Nomenclature Committee (HGNC, ^224^), OMIM Japanese translation by Gendoo ^225^, and MP ^179^. Bio-Virtuoso is expected to lower barriers to learn SPARQL using real dataset and develop SPARQL-based applications. The project has a GitHub repository ^226^.

***GNU Guix extension and deployment.*** One problem of Docker-based deployment is that it requires special permissions from the Linux kernel, which are not given in many HPC environments. More importantly, Docker binary images are 'opaque', i.e., it is not clear what is inside the container—and its state is affected by what time the container was created and what software is installed, i.e., an intermediate apt-update may generate a different image. Distributing binary images can be considered a security risk—users have to trust the party who created the image ^227^. An alternative to using Docker is using the GNU Guix packaging and deployment system ^228^, which takes a more rigorous approach towards reproducible software deployment. Guix packages, including dependencies, are built from source and generate byte-identical outputs. The hash value of a Guix package is calculated over the source code, the build configuration (inputs), and the dependencies. This means that Guix produces a fully tractable deployment graph that can be regenerated at any time. Guix also supports binary installs and does not require special kernel privileges. As of October 2016, Guix has fast growing support for Perl (473 packages), Python (778), Ruby (153), and R (277). Guix already includes 182 bioinformatics and 136 statistics packages.

At BH15, we added more bioinformatics packages and documentation ^229^ to GNU Guix and created a deployment of Guix inside a Docker container ^230^. We also packaged CWL in Guix and added support for Ruby gems to Guix which means that existing Ruby packages can easily be deployed in Guix, similar to support for Python packages and R packages. Guix comes with a continuous integration system on a build farm. We want to harvest that information to see when packages are building or failing. See, for example, the Ruby builds ^231^, which contain the SHA values of the package as well as the checkout of the Guix git repository reflecting the exact dependency graph. We are collaborating with Nix and Guix communities to get this information as JSON output so it can be used in a web service.

### Semantic metadata

***Assessing the Findable, Accessible, Interoperable, and Reusable Principles.*** Loosely defined practices in scholarly data publishing prevent researchers from extracting maximum benefit from data intensive research activities, and in some cases make them entirely unusable ^232^. There has been a growing movement encompassing funding agencies, publishers, academics, and the public at large to promote “good data management/stewardship”, and to define and enforce more stringent rules around the publication of digital research objects, including published data, associated software, and workflows, so that they are easily discoverable and readily available for reuse in downstream investigations ^233^. These include international initiatives such as the Research Data Alliance (RDA, ^234^ and ^235^), and Force11 ^236^. However, the precise nature and practice of “good data management/stewardship” has largely been up to the producer of digital objects. Therefore, bringing some clarity around the goals and desiderata of good data management and stewardship, and defining simple guideposts to inform those who publish and/or preserve scholarly data, would be of great utility.

Stakeholders in the publication of research data, including several authors of this article, participated in the development of an initial draft of the Findable, Accessible, Interoperable, and Reusable (FAIR) principles. The principles were intended to define the key desiderata for the features and/or behaviors that should exist to facilitate data discovery and appropriate scholarly reuse and citation. A public draft ^237^ was published for public comment, and BH15 participants formed a breakout group to carefully examine them against the following criteria: necessity, clarity, conciseness, independence, sufficiency, implementability and relevance. Our critical evaluation led to the development of a revised set of principles that were actionable, and improved coverage and comprehension. The text of these principles was published verbatim in a recent issue of Scientific Data ^238^. These revised principles have been widely lauded ^239^ by researchers ^240, 241^, and US and European agencies such as the National Institutes of Health (NIH, ^242, 243^) and Elixir ^244^, as being highly informative and providing insight into what it means to be “FAIR”. Future work will focus on the development of quantitative measures of adherence to the principles to assess the FAIRness of a digital resource.

***FAIR projector prototype development.*** Data discovery, integration, and reuse are a pervasive challenge for life sciences research. This is becoming even more acute with the rise of scholarly self-archiving. Much effort has been devoted to the problem of data interoperability, whether through data warehousing ^245^, ontology-based query answering ^246^, or shared application programming interfaces (APIs, ^247^). At BH15, a group of participants further developed a novel idea that was first proposed at a Data FAIRport meeting in 2014, called FAIR Projectors. FAIR Projectors are simple software applications that implement the FAIR principles by “projecting” data in any format (FAIR or non-FAIR) into a FAIR format. A projector will make use of a template-like document called a FAIR Profile, which acts as a meta-schema for the underlying data source. These meta-schemas may be indexed as a means to discover the projection of a dataset that matches the integrative requirements, i.e. the structure and semantics, of a particular workflow.

To be FAIR themselves, and thus reusable, we have selected the RDF Modeling Language (RML, ^248^), where RDF documents are used to model the structure and semantics of another RDF document. For the functionality of the Projectors, we identified an emergent, RESTful, LOD technology – Triple Pattern Fragments (TPF, ^1^), as a compelling platform that could execute the desired Projector behavior without inventing a new API. This is because TPF natively uses RDF model to publish information which can be served as a RESTful API and thus realizes a Linked Data service by nature. By the end of BH15, we had completed a prototype FAIR Projection system, and had shown how this could be integrated with other components of the nascent FAIR Data publication infrastructure. The result of this development exercise was recently published ^249^.

***Ontology metadata mapping.*** Identification of equivalent or similar concepts between vocabularies is key to the analysis of aggregated datasets that use different terminologies. Efforts such as UMLS build and maintain a system for mapping biomedical ontologies to one another. However, such mappings depend on specific versions of the ontologies, and any one version can impact scientific analyses ^250^. Therefore, having access to ontology and mapping metadata is critical to the interpretation and reproducibility of results for bioinformatics research. Initiatives such as the Open Biomedical Ontologies (OBO) Foundry ^251^, Linked Open Vocabularies (LOV, ^252^) and the National Center for Biomedical Ontology’s (NCBO) BioPortal ^253^ have put forward schemas for ontology metadata. The Ontology Metadata Vocabulary (OMV, ^254^) was first published in 2005, but does not reuse current standard vocabularies. In contrast, the Metadata for Ontology Description (MOD, ^255^) does reuse existing properties from SKOS, Friend Of A Friend (FOAF, ^256^) and Dublin Core and Dublin Core Terms (DC, DCT, ^257^).

Recently, the W3C Semantic Web for Health Care and Life Sciences Interest Group ^258^ published a computable specification for the description of datasets, which could also be applied to the description of ontologies ^259^. With respect to mappings and their metadata, SKOS offers a lightweight system for terminology mappings, while Open Pharmacological Concept Triple Store (Open PHACTS, ^260^) put forward a more detailed proposal ^261^ for mappings between RDF datasets, or LinkSets. Yet, in our experience, additional attributes are needed for both ontology and mapping metadata. Therefore, we propose an enhanced metadata scheme as a best practice for ontologies and mappings so as to improve their discovery, analyses, and reporting of results.

Our goal was to define a minimal set of attributes and standards for ontology mapping metadata. We used manually defined and automatically detected disease mappings in DisGeNET ^262^ as a case study ^263^. Our approach involved compiling attributes from the use case, identifying metadata requirements from related initiatives including ontology repositories (Ontobee, ^264^; the Ontology Lookup Service, OLS ^36^; NCBO BioPortal; Aber-OWL, ^265^), large-scale providers of mappings (UMLS, NCBO, Open PHACTS), as well as from individual ontologies including the DO ^178^, HPO ^26, 166^, ORDO ^195^, SIO ^266^, the Ontology for Biomedical Investigations (OBI, ^267^) and the Experimental Factor Ontology (EFO, ^268^). We analyzed the mapping metadata and devised a more exhaustive metadata specification for mappings ( Table 1) and ontologies ( Table 2).

**Table 1.** Exhaustive metadata for mappings.

**Attribute**	**Source**
Identifier (IRI)	FAIR
Title	Open PHACTS
Description	Open PHACTS
Publisher	Open PHACTS
License	Open PHACTS
Issued	Open PHACTS
Link to mapping file	Open PHACTS
Type of Subject	Open PHACTS
Type of Object	Open PHACTS
Type of Mapping	Open PHACTS
Link to Subject dataset metadata	Open PHACTS
Link to Object dataset metadata	Open PHACTS
Mapping relationship	Open PHACTS
Mapping justification	Open PHACTS
Authorship-who	Open PHACTS
Authorship-when	Open PHACTS
Creator-who	Open PHACTS
Creator-when	Open PHACTS
Version of mapping tool	Open PHACTS
Assertion method	Open PHACTS
Assertion value (exact, ntbt, …)	ORDO
Mapping directionality	OBAN
Mapping state (active, obsolete, other)	BioHackathon 2015
Concept overlap value (n:m)	BioHackathon 2015
Provenance/source of mapping (ontology/dictionary/database + version)	BioHackathon 2015
Evidence (PMID, Web, EHR..)	BioHackathon 2015
Curation state	ORDO
Curation author	ORDO
Curation date	ORDO
Curation justification	BioHackathon 2015
Mapping version	BioHackathon 2015
Mapping previous version	BioHackathon 2015
Link to the linkset metadata	BioHackathon 2015
Ontology version	BioHackathon 2015
Link to the ontology metadata	BioHackathon 2015
Link to mapping tool metadata	BioHackathon 2015
Sustainability (code development environment)	BioHackathon 2015

**Table 2.** Minimal metadata for an ontology.

**Metadata attributes**
IRI
Namespace
Title
Description
Format
Contact
Homepage
*Versioning*
Version
Previous version
Number of active terms
Number of obsolete terms
Number of anonymous terms
*Ontology structure*
Number of classes
Number of children
Number of property types
Number of axioms
Number of instances
Maximum depth
Maximum number of children

**Table 3.** Statistics from the EFO ontology (OWL version of date: 7th September 2015) parsed using a script in python developed during BH15 ^228^.

**Statistic**	**Count**
Number of IDs	6032
Number of ID Names	6032
Number of obsolete IDs	772
Number of active IDs	5260
Number of EFO2UMLS mappings	55
Number of IDs with UMLS mapping	52
Number of IDs without UMLS mapping	5208

Our work revealed a lack of common annotation in the description of mappings in both the attributes and vocabularies used. The inclusion of justification, provenance, evidence, directionality and versioning of mapping metadata has the potential to increase trust in the interpretation, reliability and reusability of mappings. Other provenance maintaining approaches such as Nanopublications ^269^, singleton properties, or the Ontology of Biomedical AssociatioNs (OBAN, ^270^) that could be used to model this metadata description at individual mapping level to enable a more well detailed and fine-grained semantics description. Having good quality descriptions of ontology and mapping metadata is also relevant for ontology repositories such as BioPortal and Aber-OWL, ontology and data mapping services, and for methods geared towards scientific discovery. The right vocabulary for the metadata description of mappings should be determined through wide community agreement.

***Experimental metadata representation.*** Good science must generate reproducible results ^271, 272^, and one aspect of reproducibility is the description of experimental methods and reagents used to generate the reported outcomes. Researchers write the protocols to standardize methods, to share their “know how” with colleagues, and to facilitate the reproducibility of results. Protocols typically specify a sequence of activities that may involve equipment, reagents, critical steps, troubleshooting, tips, and other essential information. Efforts such as CEDAR ^273^ and ISA-Tools ^274^ offer software and data standards to facilitate data collection, management, and reuse of experimental metadata ^275^. Ontologies such as the OBI, the SIO, and the ontology of scientific experiments (EXPO, ^276^) offer vocabulary to capture the design, execution and analysis of scientific experiments, including the protocols, materials used, and the data generated.

The Experiment ACTions ontology (EXACT, ^277^) suggests a meta-language for the description of experiment actions and their properties. The LABoratory Ontology for Robot Scientists (LABORS, ^278^) that addresses the problem of representing the information required by robots to carry out experiments; LABORS is an extension of EXPO and defines concepts such as “investigation”, “study”, “test”, “trial” and “replicate”. Finally, the SeMAntic RepresenTation for experimental Protocols ontology (SMART Protocols, ^279^) is an application ontology designed to describe an experimental protocol. The SMART Protocol framework proposes a minimal information unit for experimental protocols; the Sample, Instrument, Reagent, Objective model (SIRO, see 279), has been conceived in a way similar to that of the Patient Intervention Comparison Outcome (PICO, ^280^) model. It reuses a number of existing ontologies including the Information Artifact Ontology (IAO, ^281^), the OBI, the BioAssay Ontology (BAO, ^282^), the Chemical Entities of Biological Interest (ChEBI, ^283^), the EFO, the Eagle-i Resource Ontology (ERO, ^284^), EXACT, and the NCBI taxonomy ^285^. Semantic Web technologies including ontologies and Linked Data enable semantic publication of experimental protocols, their classification, and the mining of textual descriptions of experimental protocols.

Limitations of current approaches to experimental metadata include an inability to cover the “digital continuum”—from the highly diverse set of complex processes in laboratories to the needs expressed by regulatory affairs. There also lacks a rapid mechanism to add new concepts into existing ontologies and terminologies. Finally, experimental information is often scattered over a complex network of applications ranging from Laboratory Information Management Systems (LIMS) to text processors and Excel spreadsheets and, most of all, laboratory notebooks. Researchers keep a detailed description of their daily activities, results, problems, plans, derivations of the original plan, ideas, etc. in their laboratory notebooks.

**Figure 6.**
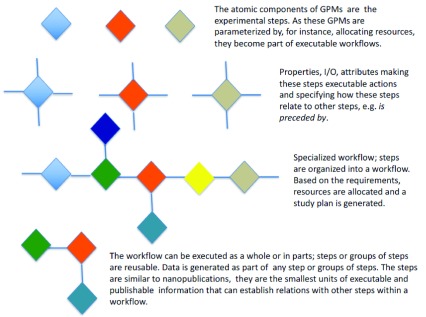
From a General Process Model to an executable workflow.

As a high-level abstraction serving to represent laboratory workflows, we argue, a General Process Model (GPM) is needed. GPMs often represent a networked sequence of activities, objects, transformations, and events that embody strategies for accomplishing a specific task. Such models can be instantiated and specialized as needed. Figure 6 illustrates how a GPM could be further instantiated. The model starts by defining actions in a laboratory. These should be generic so that they can be made concrete as specifics from the laboratory are added, e.g. properties, inputs, and outputs. These generic objects can be linked in terms of inputs and outputs. Once there is an abstract workflow, resources are then allocated. The execution of the workflow instantiates all the properties for each object; data is thus generated with rich, process-related metadata. Repositories such as Dryad ^286^, FigShare ^287^, Dataverse ^288^, and many others structure metadata primarily for describing generic attributes of the datasets while more specialized repositories such as the Gene Expression Omnibus (GEO, ^289^) or the PRoteomics IDEntifications database (PRIDE, ^290^) capture specific elements of the experimental record. Our work to develop a GPM will provide a basis by which published data, metadata, and the experimental protocols used will establish a mechanism by which researchers may execute data sharing plans that meet the expectations of funders, journals and other researchers.

***Knowledge graph annotation for human curation.*** Manual curation of biomedical repositories is a well-established practice in the life sciences domain to improve the accuracy and reliability of data sources. An increasing number of repositories is being made available as networks of concepts and relations, i.e. “knowledge graphs”. Currently, a tool or data source that exposes (part of) a knowledge graph typically provides an annotation facility to allow curators (or the general public) to make or suggest changes. However, such annotations are often only used within the context of that particular tool, for example to notify curators that there may be a problem with a certain data entry, but frequently remain unusable and undiscoverable for other purposes.

For this reason, we have developed a tool called the Open, Reusable Knowledge graph Annotator (ORKA, ^291^). ORKA is a small, embeddable web service and user interface to capture and publish an annotation event. A typical workflow looks like this:

1. A user or curator of a graph-based resource wants to report a defect or comment on a particular edge of the graph.
2. The resource provides a link that forwards the user to the ORKA user interface.
3. The user is identified by means of one of several open authentication options.
4. The user may now “edit” or comment on the particular graph edge.
5. The annotation is captured and stored and the user will be redirected to the interface of the original resource.

ORKA aims to support annotation from a wide range of data sources and tools that are either based on or can be mapped onto a knowledge graph. ORKA can be integrated with such resources by enabling a request to its API. In a user interface this may look like an “Annotate now” link, button, or context menu item, on an association or assertion from the knowledge graph. The API requires minimally a pointer to the original data source and the selected knowledge graph assertion specified as a single triple, i.e. RDF URIs for subject, predicate and object. In subsequent steps, ORKA will collect the identity of the user and record the annotation activity as a self-contained, semantically interoperable digital object.

To identify the user, we envision a choice from a range of commonly used open authentication identity providers. In the current prototype, Open Researcher and Contributor ID (ORCID) provides the main method of user authentication. We consider the identity of the annotator to be an essential part of the provenance of the annotation: firstly, it can be used to rate or establish trust in the quality of a curator and, secondly, it is needed to reward proper credit to the user for their curation effort.

In the annotation stage, the user currently has the option to change the relation, add a comment, or both. We found that offering only a limited set of annotation options helps keep the annotation process quick and simple, yet still expressive. Through the selection of an alternative relation, the user suggests an improvement that includes using a more specific predicate or negating the relationship. Relations may be chosen from a pre-loaded set, or from a specific ontology chosen by the user. The free text box can be used to make any additional comments, and currently also serves as a catchall to describe any other type of annotation: for example, to support an assertion with additional evidence, or when a suitable predicate is not readily available.

Finally, ORKA captures the annotation, including provenance information (curator ID, date, original source triple and context), as a semantic digital object using the Nanopublication ^269^ model and the Open Annotation ontology (OA, ^292^). The annotation object is then stored in an annotation repository, which is by default an open Nanopublication store (ORKA can also be reconfigured to store to a private location). Subsequently, the user has the option to browse the repository or return to the original resource from which the annotation request to ORKA was made. Meanwhile, the original data source will receive a notification and link to the annotation object. Data sources may then apply different strategies to incorporate the annotations in their resource: some may first want to perform manual validation, or choose to accept annotations from a selected group of annotators automatically. We note that the semantic description of the annotations and their provenance promotes the reuse of annotations: third parties can access the (public) annotation stores and use them for their own purpose. Attribution can be achieved, as Nanopublications are inherently citable.

We have designed ORKA as a generic service to annotate different types of graph-based data sources and produce persistent, reusable semantic digital annotation objects. During BH15 we developed a browser bookmarklet that enables annotation of any web page with embedded RDFa statements ^293^. ORKA is currently being developed in the context of the ODEX4All project ^294^ to enable annotation of its core knowledge platform. Initial use cases have suggested a need for additional features, such as annotation of the object of a statement as well as specifying evidence for an annotation (for example by citing published literature). Supporting additional open authentication methods will lower the entry barrier for potential users even further. In the future, we hope to integrate ORKA in other resources and work out scenarios to show how generically reusable annotations result in richer, more accurate data sources and how this helps knowledge discovery in the life sciences domain.

## Conclusions

The BioHackathon series offers an unparalleled opportunity for scientists and software developers to work together to tackle challenging problems in the life sciences. BH15, the 2015 edition, was no exception, and featured contributions from a wide range of subdisciplines.

On the topic of *semantic metadata*, we observed the FAIR principles gaining further traction with the development of additional tooling in the form of FAIR Projectors that represent data in FAIR ways using a template-like system. Likewise pertaining to semantic metadata, work was done at BH15 to assess the state of the art in recording the justification, provenance, evidence, directionality and versioning of ontology mappings. Additionally in this track, participants initiated work on a General Process Model to capture lab experimental metadata as networked sequences of activities, objects, transformations, and events. Lastly in semantic metadata, participants worked on the ORKA system for annotating knowledge graphs by human curators. To contribute to the improvement of *reproducibility* in bioinformatics, participants in that track worked on three technologies that formally represent the steps of *in silico* experiments and the computational environment in which such experiments take place. The Common Workflow Language (CWL) is a system to describe command line tools and chain them together. At BH15, participants added CWL support to the Toil workflow engine and worked on CWL components that consume JSON(-LD). In addition, the Docker lightweight system for virtualization (‘containerization’) was targeted at BH15 to enable discovery of bioinformatics containers and simplify deployment of the Virtuoso triple store loaded with bioinformatic data sets. Lastly contributing to reproducibility, participants further extended the GNU Guix ecosystem, an alternative approach for virtualization with certain security advantages, by adding additional bioinformatics packages as well as CWL and Ruby gems to it.

In the track on *genotypes and phenotypes*, participants worked on the semantic representation of genotype and phenotype data. This included the modeling of common, stably named and canonically identifiable genomic variation as an RDF graph that was queried using SPARQL. Conceptually related to this, other participants worked on a real-time generated, queryable, semantic representation of VCF data, a commonly used format for representing variant calls such as SNPs. Contributing in this track to the semantic representation of phenotypes, participants worked on the translation of the Human Phenotype Ontology in Japanese. In efforts to contribute to the representation of comparative data within frameworks of shared evolutionary ancestry, participants in the *orthology and phylogeny* track focused on two challenges. Firstly, work was done on the development of the Orthology Ontology to capture essential concepts pertaining to sequence orthology, including evolutionary events such as sequence duplication and speciation. Secondly, to attempt to place such evolutionary events on absolute time scales, an evaluation was made of the amenability of the FossilCalibrations database on the semantic web by implementing a prototype pipeline that calculates substitution rates for branches between speciation events as a function of time since gene duplication.

Contributing to semantic representations in *chemistry*, participants discussed strategies to advance cooperation between chemical databases, including establishing agreement on which database keys to use, how to make databases more interoperable by denser cross-referencing, and harmonizing RDF representations. Also in this track, more work was done on the PIERO ontology for chemical transformations, including improved RDF interoperability and additional data curation. An important development was the definition of a classification based on reaction characteristics. Moving on to larger molecules, in *proteomics*, participants assessed the scalability of representing the UniProtKB database in OWL. Other participants in the same track worked on ontologizing proteome data. An important resource in this field is jPOST, for which an assessment of available controlled vocabularies and ontologies took place and work on an RDF schema commenced. In *glycomics*, participants worked on extending the development of an ontology for representing glycan structures, GlycoRDF, which was initiated at an earlier BioHackathon, in 2012.

In *metabolomics*, participants worked on improving the availability on the semantic web of data pertaining to the biochemical analysis of low-molecular-weight metabolites in biological systems. This included a focus on the visualization of plant metabolome profiles and the identification and annotation of metabolites. Participants in this track further worked on the development of visual web applications to expose the metabolome database AtMetExpress.

In the *natural language processing* track, participants worked on the capture and analysis of human phenotype data from free form text, i.e. the biomedical literature. Other participants in the same track worked on mining the structured text from drug labels to collect rich representations of drug (side) effects and indications. Also in this track, work was done on data analytics on text-mined corpora, specifically to attempt to learn associations between biological processes disrupted by gene mutations with externalized phenotypes. Large assessments were made of the integration of text mined and curated data and of the interoperability of disease terminology. As a demonstration of the state of the art in generating natural language, a demo was developed that generates a haiku from data on the semantic web.

The *data retrieval and query answering* track was concerned with new technologies for interrogating data on the semantic web. This included exposing semantic web services for OpenLifeData through re-implemented, more scalable interfaces. Participants in this track also worked on the SPARQL Builder, a tool for more easily constructing queries in the commonly used, but not very user-friendly, SPARQL language. Other ways to make queries easier included work on LODQA, a system that constructs queries from natural language. A final demo of the state of the art in interrogating the semantic web in a playful way was Crick-Chan, which presents itself through cartoon animations and interacts with users through a chat bot interface.

BH15 thus contributed to many challenges in bioinformatics, including the representation, publication, integration and application of biomedical data and metadata across multiple disciplines including chemistry, biology, genomics, proteomics, glycomics, metabolomics, phylogeny and physiology. A wealth of new semantics-aware applications have been developed through this hackathon that facilitate the reuse of complex biomedical data and build on global efforts to develop an ecosystem of interoperable data and services. As requirements for providing higher quality data and metadata continue to grow worldwide, BioHackathon participants will be well positioned to develop and apply semantic technologies to face the challenges of tomorrow.

## Data availability

No data are associated with this article.

## Software availability

**Version control repositories to which code was committed during the BioHackathon are aggregated at:** https://github.com/dbcls/bh15/wiki/Hackathon-source-code-repositories.

**Archived code at time of publication:** https://doi.org/10.5281/zenodo.3634405 ^62^.

**License:** Creative Commons Attribution 4.0 International.
