# Supplementary material for: BioHackathon 2015: Semantics of data for life sciences and reproducible research
Source: F1000Res. 2020 Feb 24;9:136. [Version 1] doi: 10.12688/f1000research.18236.1 (PMC7141167; doi:10.12688/f1000research.18236.1)
Supplement: Peer review history

# Peer review: Reviewer response for version 1

**DOI:** 10.5256/f1000research.19948.r60451

**Published:** 2020-04-07

**Authors:** James P. Balhoff, Gaurav Vaidya

**Affiliations:** 1 Renaissance Computing Institute, University of North Carolina at Chapel Hill, Chapel Hill, NC, USA

This is a sprawling and fascinating report from a hackathon in 2015. The activities involve development of software and standards across a broad range of subdisciplines within the life sciences, but are united by largely basing their approaches on the application of semantic technologies. As such, the paper is an excellent overview of a wide variety of ways in which semantic technologies are being employed with great success in the biological sciences. Areas targeted include genotype/phenotype data, orthology and phylogeny, proteomics, metabolomics, data retrieval and querying, natural language processing, reproducibility, and metadata representation. Despite the several years that have elapsed since then, many of the tools built and insights gleaned still have relevance today, and so we are grateful that this work has been written and published. However, we believe that this five year gap between event and publication provides a valuable opportunity for the authors to reflect on how that hackathon work was useful, both in its original context as well as today. We think the authors did an excellent job describing this in the section “Assessing the Findable, Accessible, Interoperable, and Reusable Principles”, but some other sections suffer by not being clear about what was developed before, during and after the hackathon. It may turn out that some of this work was not particularly useful in the long term — which is only to be expected in a hackathon — but it might also be that a good idea from five years ago has been subsequently overlooked, and this work might be in a position to call attention to such ideas. 

 Having explicit subsections entitled “Changes in the landscape since 2015” in every section could be helpful in making this clearer. There are also several references to events that were contemporaneous with the hackathon; for example, in the section “Molecular evolutionary process calibration”, the authors write that “Recently, a working group at the National Evolutionary Synthesis Center (NESCent) …”. However, this is incorrect to state in a document published in 2020, as NESCent was shut down in June 2015. Clarifying which parts of the text are true as of the workshop and which are true today would help to prevent such errors.

 Understandably for activities at a hackathon, some activities could be described in a little more detail, while some descriptions could be more concise. Some notes on the various descriptions follow:

 Variation graph construction:

- It would be helpful to state which triplestore database was used when stating performance results.

Orthology ontology development and application:

- Grammatical issue in sentence including “Although the standard mapping and transformation by SWIT was largely able to transform the content of the three databases, though a few resource-specific rules were necessary because” (remove ‘though’?).

Molecular evolutionary process calibration:

- The authors point out that a particular JSON resource is “convenient for programmers but it also means that certain concepts used in the JSON are ambiguous as they are not linked to any controlled vocabulary or ontology.” They should mention JSON-LD as a possible solution to this problem, a technology they refer to several times elsewhere in the paper.

Protein semantic representation:

- It would be useful to include a brief statement about how the particular axiomatization described supports some use cases. Besides OWL 2 EL scalability, what motivated this particular design?
- "For complex datasets, the additional semantics of OWL, which includes assertions of disjointness, i.e. the explicit semantic distinction between classes and their instances, and axioms restricting the use of classes and object properties, may be particularly beneficial." Here, it seems like an inaccurate definition is provided for ‘disjointness’ (“explicit semantic distinction between classes and their instance”). This is not what disjointness means, but perhaps this was just meant to be a list of logical features provided by OWL. Remove ‘i.e.’?
- A layout issue we noted was the sentence “We also generate the following axioms (here expressed in Manchester OWL Syntax)”, which should not be a bullet point.

Tools for metabolite identification and interpretation / Plant metabolome database development:

- These sections provide a large amount of background information, taking longer to reach the description of what was accomplished at the hackathon.

Chemical database integration:

- Perhaps it wasn't discussed at the hackathon, but we are curious how the ChEBI ontology fits into this picture of harmonization across chemical databases.

Clinical phenotype text mining:

- The superscript citation format makes some of the sentences oddly worded (e.g., second paragraph) where it seems like the author name is meant to be in the sentence. "For example ^160^, assessed the contribution of …”. In this case, it appears that the citation was intended to be included inline, i.e. “For example, Kotfila and Uzuner (2005) assessed ...”. In these cases, the text should be rewritten so that it is easier to read. 

The abbreviation section could benefit from hyperlinks to the ontologies (and possibly also the organizations) being linked to.

 Apart from these relatively minor issues, we are grateful that the authors have published this work and recorded the activities at what appears to be a wide-ranging and productive hackathon. Also, congratulations to them on producing the first semantic haiku!

We confirm that we have read this submission and believe that we have an appropriate level of expertise to confirm that it is of an acceptable scientific standard.

# Peer review: Reviewer response for version 1

**DOI:** 10.5256/f1000research.19948.r60449

**Published:** 2020-03-31

**Authors:** Jeremy G. Frey

**Affiliations:** 1 School of Chemistry, Faculty of Engineering and Physical Sciences, University of Southampton, Southampton, UK

This is a well written report of a Hackathon activity in the area of semantics and ontologies for life sciences with a view to using these semantic technologies and knowledge structures to enhance the reproducibility and I would say the use and re-use of life sciences data and methods in a number of typically gene and protein related areas. While the report is on an activity which I presume took place in 2015 the material is very relevant and not dated.  

 The report contains very good summaries of the prior art on the areas, especially ontologies that cover the areas of interest and are of potential use across the whole of the scientific research life cycle. The details of the challenges, data made available, teams and goals are provided and will benefit the community though it will need detailed reading to absorb the extensive material provided by the authors. The extensive references are themselves a very useful research source.

 I consider this to be a well written and useful contribution to the literature which will help the community in building new and useable systems (and also not to re-invent things that do already work).

I confirm that I have read this submission and believe that I have an appropriate level of expertise to confirm that it is of an acceptable scientific standard.
